# Recent Progress of Electrospun Herbal Medicine Nanofibers

**DOI:** 10.3390/biom13010184

**Published:** 2023-01-16

**Authors:** Hang Liu, Yubin Bai, Chang Huang, Ying Wang, Yuexin Ji, Yutong Du, Lin Xu, Deng-Guang Yu, Sim Wan Annie Bligh

**Affiliations:** 1 School of Materials and Chemistry, University of Shanghai for Science and Technology, Shanghai 200093, China; 2 School of Health Sciences, Caritas Institute of Higher Education, Hong Kong 999077, China

**Keywords:** herbal medicine, biopolymers, electrospinning, nanofibers, drug delivery, wound dressing, tissue engineering, food packaging

## Abstract

Herbal medicine has a long history of medical efficacy with low toxicity, side effects and good biocompatibility. However, the bioavailability of the extract of raw herbs and bioactive compounds is poor because of their low water solubility. In order to overcome the solubility issues, electrospinning technology can offer a delivery alternative to resolve them. The electrospun fibers have the advantages of high specific surface area, high porosity, excellent mechanical strength and flexible structures. At the same time, various natural and synthetic polymer-bound fibers can mimic extracellular matrix applications in different medical fields. In this paper, the development of electrospinning technology and polymers used for incorporating herbal medicine into electrospun nanofibers are reviewed. Finally, the recent progress of the applications of these herbal medicine nanofibers in biomedical (drug delivery, wound dressing, tissue engineering) and food fields along with their future prospects is discussed.

## 1. Introduction

Herbs are widely used in traditional medicines, such as traditional Chinese medicine (TCM), Indian medicine (Ayurveda, Unani, Xida), Japanese Kampo, Korean medicine, etc. It has been used for hundreds or even thousands of years [1,2,3]. Compared to “one disease, one target and one drug” mode of delivery and treatment of synthetic drugs, herbal medicine contains a variety of components which can play a multi-target synergistic effect [4]. The bioactive components of traditional herbal medicines mainly include phenols, saponins, flavonoids, tannins, terpenoids and alkaloids. They have antibacterial, antiviral, anti-inflammatory, antioxidant, anti-tumor, analgesic, immune regulation and tissue regeneration effects [5]. A detailed summary is given in Table 1. In addition, a personalized prescription is provided by adjusting the specific medication for each patient depending on the syndrome [6]. It is precisely because of these characteristics, TCM offers a good therapeutic effect in the fight against the global epidemic of novel coronavirus (COVID-19) [7,8].

Although traditional herbal medicines have been used for centuries and have a good effect in fighting diseases, they also have drawbacks such as poor solubility of bioactive compounds and poor permeability of biofilms, which makes the bioavailability of plant components poor [64]. Traditional drug delivery systems, such as extracts, oral liquids, granules, powders, and capsules, cannot overcome these problems. The development of nanotechnology has enhanced the bioavailability of these bioactive compounds and jointly promoted safe, effective and convenient drug delivery systems [65]. At present, the technology of manufacturing nanofibers includes stretching [66], self-assembly [42], phase separation [67], template synthesis [68], physical and chemical vapor deposition [69], electrochemical deposition [70], laser ablation [71], freeze-drying [72], solvent casting [11], solution blowing [73], dry-wet spinning [74], force spinning [75] and electrospinning [76]. Among them, electrospinning is a branch of electrohydrodynamic atomization (EHDA). Its process is simple, low cost, high efficiency and reproducibility, and a wide range of raw materials. Continuous nanofibers with diameters from micron to nanometer can be manufactured by optimizing process conditions.

**Figure 1:**
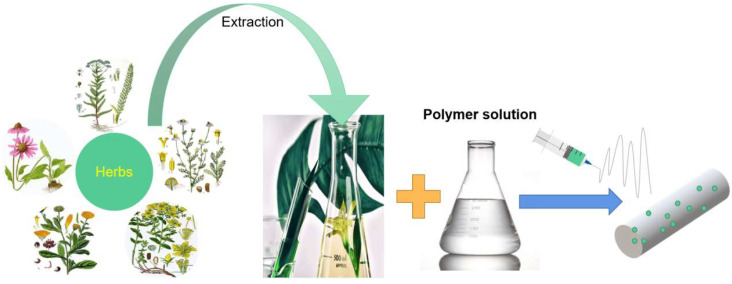
Fabrication process (plant image for reference), reprinted with permission from [77] copyright 2020, Springer Nature.

Synthetic and natural polymer materials are critical to the fabrication process (Figure 1). They determine the activity and delivery mechanism of herbal medicines. Most of the natural polymers are biocompatible, nontoxic, biodegradable, and versatile. Versatility is reflected in its extracellular matrix (ECM)-like properties that promote cell proliferation and adhesion. It can also be used for scaffold construction to provide an ideal environment for cell growth and proliferation. However, they have some limitations, such as poor mechanical properties, uncontrolled rapid degradation, and the possibility of pathogen contamination. In order to overcome these problems, some biological synthetic polymers have been considered for their uniform quality, flexibility in synthesis, processing and modification, and their good mechanical stability in vivo. However, these biological synthetic polymers are ”coated” to ensure their controllability and target delivery capabilities. Hence a combination of natural and biological synthetic polymers can promote better cell adhesion and proliferation by retaining the biological properties of natural polymersto give good mechanical properties, durability and controlled biodegradability.

**Figure 2:**
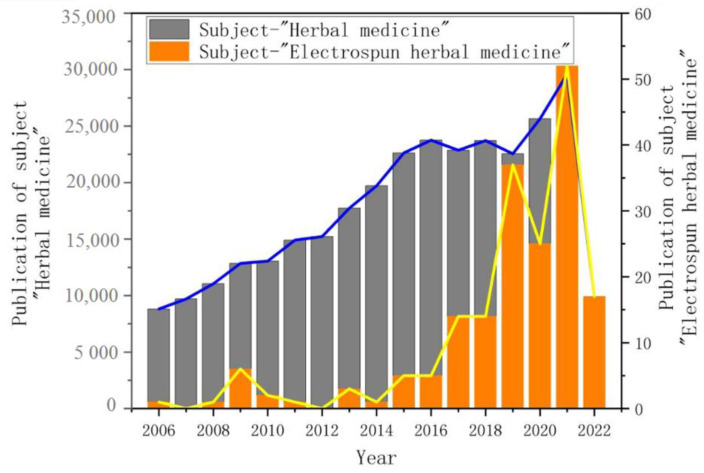
Statistics of literature retrieval from the “Web of Science” with keywords search on “Herbal Medicine” and “Electrospun Herbal Medicine”.

In the Web of Science, we searched for the number and trends of articles published on “Herbal Medicine” and “Electrospun Herbal Medicine” over the past 10 years, as shown in Figure 2. Herbal medicine related research is up to ten thousand. However, the total number of research articles on “electrospun herbal medicine” is not large. Since the outbreak of the new coronavirus epidemic, the number of articles has surged and shown a steady increase compared with the previous years. This shows that the use of electrostatic spinning technology in drug delivery is no longer reserved for single compounds. This review aims to explore the latest progress of electrospun herbal medicine nanofibers in drug delivery, wound healing, tissue regeneration and food packaging applications. The application development of electrospinning technology and various polymer materials for encapsulating herbal medicine are discussed, respectively.

## 2. Electrospinning Technique

### 2.1. Introduction of Electrospinning Technology

Electrospinning (ES) is a branch of electrohydrodynamic atomization (EHDA) which can be used to produce morphologically controlled single-layer or multi-layer fibers (1D-3D multi-dimensional structures or complex nanostructures) at the micron and nano scales by modifying polymers, solvents or using additives such as surfactants and crosslinking agents [18,22]. At the same time, the electrospinning process does not generate heat in use, which is very important for maintaining the structural components of bioactive substances during processing and storage [78,79,80]. Due to its simplicity, low cost, easy scalability, flexibility of alternative materials and operation, and high efficiency, electrospinning has its advantage in manufacturing nanofibers [79].

**Figure 3:**
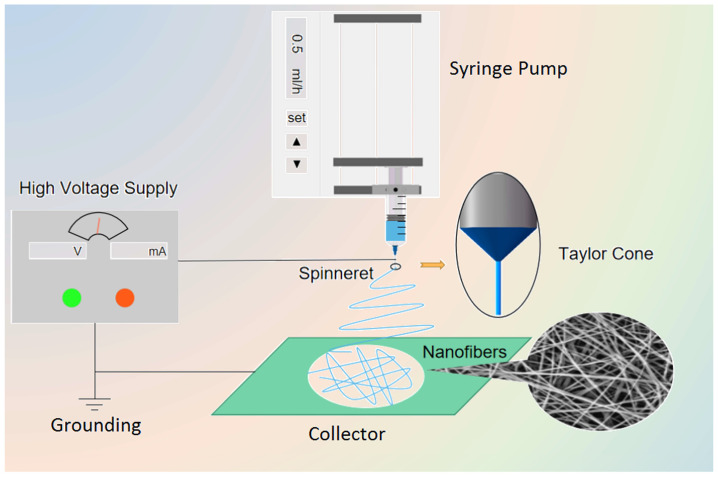
Diagram of electrospinning device.

Electrospinning technology is a process of ejecting charged liquid jet from liquid surface by using high-voltage power to overcome viscous polymer solution. The high-voltage power supply connecting the needle and the metal collector will provide a high electric field. Under the application of the high-voltage electric field, the spinning solution (solution, suspension, melt) [81] pulls out the continuous fluid through the needle at a stable flow rate, and the resulting micro/nanofibers are randomly collected onto the grounded collector [82]. The basic device of electrospinning includes five components (Figure 3): (1) high voltage power supply running in the range of kV; (2) Injection pump for measuring working fluid; (3) syringes containing polymer solution; (4) Metal spinning needle (single or structure); (5) Fiber collector. The basic principle of this technology is to access a voltage, which is input through a conductive needle. Driven by the injection pump, the high-voltage power supply applies an electrostatic force to the spinning solution from the syringe to the needle due to the presence of various forces and charges acting on the solution. When the electrostatic force overcomes the surface tension of the fluid, the spinning droplets deform at the tip to form a “Taylor cone “. When the force exceeds the critical value of the surface tension of the conical droplets, a jet will be formed. At the same time, the volatile solvent in the spinning solution evaporates (drying process), and long and thin filaments are ejected from the tip. These filaments solidify and then deposit on the grounded collector to form uniform nanofibers [41,78].

The characteristics of electrospun nanofibers are small fiber diameter, high surface area, high porosity, elasticity, and easy functionalization. Nanofibers formed by natural and synthetic polymers possess high mechanical properties, high encapsulation efficiency, sustained release properties, biocompatibility, biodegradability, low toxicity, and structural similarity to the extracellular environment of tissues. These properties can support cell synthesis of specific proteins and other biochemical and biological processes required for healthy tissue growth [28,83]. Therefore, electrospun fibers have become excellent candidates for various applications, such as catalysis [84], filtration [85], water treatment [86,87,88,89], biosensors [90,91,92], food packaging [93,94], cosmetics [95] and biomedicine [96,97,98,99,100,101].

Especially in the field of biomedicine, electrospun fibers can be used as scaffold materials for cell/tissue culture [102,103], wound dressing materials [57,104], and carriers for local/dermal/transdermal drug delivery [57,76,105]. Nanofibers can also be important for targeted therapy in medical applications because of their high encapsulation characteristics which can protect loaded anti-inflammatory, antibacterial, antioxidant or anticancer drugs (traditional Chinese medicine and Western medicine), bioactive compounds (extracts), growth factors, small molecules, proteins, DNA, etc. [33,76,106]. It is also possible to control the physical and chemical properties (hydrophilicity, fiber diameter, density) of the fibers by changing their composition, such as blending of different polymers (natural or synthetic), to steadily release active compounds quickly or continuously [13,18,82]. They are suitable for the development of dressings and tissue scaffolds for wound healing because they can promote cell migration, adhesion, proliferation and differentiation, allowing nutrients to diffuse into the cell structure, while waste is diffused from the cell structure, allowing drug molecules to spread more easily from the matrix to achieve effective wound healing, gas to permeate, exudate to diffuse and cell respiration. In addition, they help to regulate wound moisture, prevent wound drying, and enhance tissue regeneration [107,108]. Nanofibers can be used as appropriate scaffolds to promote cell attachment and growth by creating a three-dimensional ECM to improve damaged tissue and repair organ function [43]. Some studies have shown that nanofibers can be used for bone transplantation [56], neuronal repair [50], skin regeneration and other tissue regeneration [109].

### 2.2. Classification

With the rapid development of technology, electrospinning is improved and developed in three directions: (1) From a single fluid process to coaxial [110,111], triaxial [112,113], side by side [114,115] and the combination of multi-fluid processes [116,117,118], improve the practical functions of the designed complex nanostructures; (2) the scale-up fabrication of nanofibers for realization of their medical and material applications [77,119]; and (3) direct functional application of nanofibers [120].

**Figure 4:**
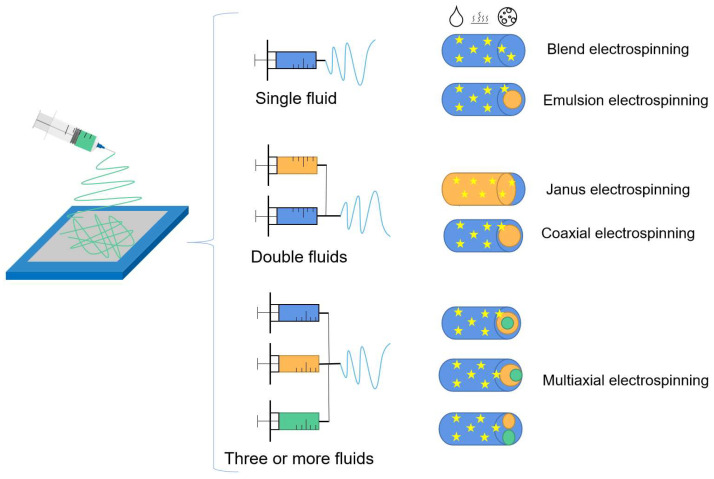
Electrospinning process classified by spinning head structure.

According to the number of spinning solution, it can be divided into single-fluid electrospinning, two-fluid electrospinning and multi-fluid electrospinning. According to the type of spinning solution, electrospinning can be divided into mixed electrospinning, melt electrospinning, emulsion electrospinning and suspension electrospinning (single fluid as an example). According to the spinning head structure, two-fluid and multi-fluid electrospinning can be divided into coaxial electrospinning and Janus electrospinning, as shown in Figure 4.

#### 2.2.1. Single-Fluid Electrospinning

In blend electrospinning, biomolecules/drugs are mixed with the polymer solution prior to the spinning process [121]. Aksit et al. [122] selected PLGA/gelatin polymers, and added different amounts of *Hypericum capitatum* var. *capitatum* (HCC) extract (a traditional herb) to prepare antibacterial membranes. The increase of HCC concentration led to the decrease diameter of the fiber and improved the hydrophilicity and degradation rate of the membrane. It also exhibits antibacterial, non-toxic, biocompatibility and cell adhesion. Marina et al. [123] loaded bioactive glass (BG) particles into PGS/PCL polymer to prepare a homogeneous bead-free composite fiber. The presence of BG particles induces an increase in diameter of the fiber, and the release of therapeutics from the composite fibers for wound healing. There are drawbacks from the simple blend electrospinning method because of the limited choice of non-toxic and environmentally friendly spinnable solvents which allow as the available polymer concentration and the incorporation of active ingredients and other additives to form composite nanofibers [124].

The melt electrospinning system is essentially the same as solution electrospinning, with an additional heating device for heating the polymer to a molten state [125]. The advantage of this process is that it does not require toxic organic solvents and can assist insoluble polymers to dissolve in solvents [126]. It is precisely because of the extra heating conditions that only thermoplastic polymers can be selected, and heat-sensitive substances cannot be added. At the same time, because of the surface charge density of the polymer melt is low, the average diameter of the fiber is large, and the cooling solidification problem of the outflow nozzle will hinder the jet stretching. Deng et al. designed a novel melt spinning device without injection pump by changing the parameters to reduce the viscosity of the polymer melt to produce a fiber with a diameter of less than 15 μm and a smooth surface [127].

Emulsion electrospinning is a kind of electrospinning of W/O emulsion. The drug is usually dissolved in the aqueous phase and then dispersed in an organic polymer solution (oil phase) containing a suitable surfactant. Through the action of high voltage current, the oil phase and the aqueous phase emulsion can be elongated and converted into a core-shell structure fiber [128]. This technique overcomes the introduction and controlled release of hydrophilic bioactive molecules from hydrophobic polymer nanofibers [129,130,131]. Shin et al. [131] prepared oil-in-water emulsion with a core of plant fungicide (oil phase)/shell of PVA aqueous solution (water phase) by one-step encapsulation through emulsion electrospinning to prepare core/sheath structure nanofibers. The prepared fibers were good (250–350 nm). The results of sterilization experiments and release curves proved the strong antibacterial activity and sustained release. Although emulsion electrospinning has many advantages, especially for drug encapsulation and thus changing drug release. However, toxic organic solvents are still used as a continuous phase (W/O emulsion) or as a separation phase (O/W emulsion), and polymer aqueous solution electrospinning also requires crosslinking to improve its stability [124]. Suspension electrospinning is introduced to achieve electrospinning from water suspension, which is called “green electrospinning”. It overcomes the limitations of electrospinning toxic solvents and high concentrations. Sun et al. [132] prepared a high solid content aqueous suspension of poly(hexamethylene adipate) PHA-b-PEO by secondary suspension/solvent replacement method, and processed it into water-stable polyester nanofibers by electrospinning of these suspensions. The highest solid content of the suspension was 16wt%, and the average particle size was 108 nm. The resulting fiber will not disintegrate in short-term contact with water and does not contain any additives.

#### 2.2.2. Double-Fluid Electrospinning

**Figure 5:**
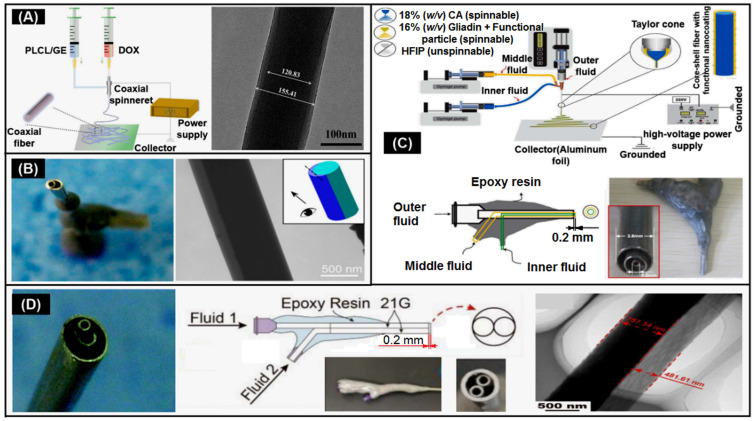
Electrospinning process for fabrication of complex structured nanofibers. (**A**) Two-fluid coaxial electrospinning process and core-shell fiber TEM diagram, reprinted with permission from [147] copyright 2022, Society of Chemical Industry. (**B**) Two-fluid side-by-side eccentric structure and TEM images of Janus nanofibers [63], reprinted with permission from [63] copyright 2018, American Chemical Society. (**C**) Three-stage coaxial electrospinning process and needle structure, reprinted with permission from [152] copyright 2021, Elsevier. (**D**) SEM images of two kinds of needles and Janus nanofibers with parallel multi-chamber structure [148,153], d1 reprinted with permission from [153] copyright 2017, Chemical Communications; d2 reprinted with permission from [148] copyright 2022, Pharmaceutics.

Double-fluid electrospinning includes both coaxial electrospinning and side-by-side electrospinning, which can be exploited for creating core-shell and Janus nanostructures, respecyively [133,134,135,136,137,138,139,140,141,142,143,144,145,146,147,148,149,150,151,152,153]. Coaxial electrospinning is carried out by two concentrically arranged conductive needles, and polymer solutions with different characteristics are injected simultaneously to adjust the parameters to produce fibers with good core-sheath layers (Figure 5A) [147]. The advantage of the core-shell structure is the ability to encapsulate drugs [133,134], bioactives [135,136,137], growth factors [138], small molecules [139], etc. into the core and shell in one step, and has better mechanical properties and controllable release [140]. This method overcomes the shortcomings of burst effect, small loading amount and activity failure of hybrid electrospinning [141,142,143]. Yang et al. [141] prepared a flexible and cell-compatible poly (glycerol sebacic acid) PGS/POLY-L-lactic acid PLLA fiber scaffold with a core-shell structure by coaxial electrospinning. The shell PLLA is used as a porous skeleton on the fiber surface, and the fiber exhibits strong skin wound tissue repair ability.

In contrast with the core-shell structure, the Janus structure enables two compartments to be in direct contact with their environment, which can produce multifunctional nanomaterials [144]. In order to resolve the repulsion and separation during the spinning process, Yu et al. [63] reported a self-made eccentric spinning head (Figure 5B), which nested a smaller metal needle in the inner wall of another metal needle tube. This design is more in line with the behavior under an electric field, as the two working fluids are side by side and their interfacial interactions are greatly reduced, facilitating successful two-fluid electrospinning. Yang et al. [145] prepared a Janus wound dressing composed of polyvinylpyrrolidone-ciprofloxacin (PVP-CIP/EC-AgNPs) ethyl cellulose-silver nanoparticles by this eccentric spinning head process showing effective drug release and antibacterial properties in in vitro studies. Xu et al. [146] designed a wound dressing with a three-layer composite intermediate layer (PCL/gelatin) Janus nanofiber containing ciprofloxacin CIP and ZnO nanoparticles. In this structural design, it not only avoids cell poisoning caused by ZnO nanoparticles contacting the skin, but also provides rapid initial drug release and sustained synergistic antibacterial effect at the wound site.

#### 2.2.3. Multi-Fluid Electrospinning

Multi-fluid electrospinning can be used to prepare multilayer or multi-chamber nanostructures. Each layer or chamber can have independent composite properties with different applications [149,150]. Yang et al. [151] developed a modified three-axis electrospinning process, using electrospinning core solution, the middle and outer layers do not have spinning conditions, to improve the quality of the core-shell structure, and adjust the concentration of the middle layer fluid to achieve the characteristics of controlling drug release. Zhao et al. [152] developed a degradable antibiotic film using modified three-stage coaxial electrospinning technology (Figure 5C), which can be used for sewage treatment. Wang et al. [148] used a self-made two-compartment parallel spinning head as shown in Figure 5D, using cellulose acetate (CA) and polycaprolactone (PCL) as polymer carriers for silver nanoparticles (AgNPs) and lavender oil (LO), respectively, and electrospun them into Janus fibers. The Janus structure on both sides of the layer can be clearly seen from the SEM, which also confirmed the successful loading of antibacterial drugs and the good antibacterial effect.

### 2.3. Influence Factors

As an electrohydrodynamic method, electrospinning is very sensitive to working parameters and affects the porosity, pore size and fiber diameter of nanofiber scaffolds. Working parameters include solution parameters, process parameters and environmental parameters. The solution parameters include (polymer molecular weight, surface tension, conductivity, concentration, viscosity), process parameters include (applied voltage, flow rate, distance between collector and tip), environmental parameters include (temperature, humidity) [154,155,156].

For the influence of solution parameters, the molecular weight of the polymer will affect the viscosity, concentration, dielectric strength, surface tension and other parameters. The molecular weight of the polymer is low, which will produce beads. As the molecular weight increases, smooth fibers are formed, and more than a certain molecular weight will form micro-strips. The surface tension of the solution is small, and continuous fibers can be produced. If the surface tension of the solution is too large, the jet will be unstable. If the conductivity of the solution is low, the fiber diameter decreases, resulting in the formation of uneven fibers and beads. When the solution concentration or viscosity is too low, beading is formed, and when the concentration/viscosity is too high, discontinuous fibers and beading are also produced because the solution has poor fluidity and is difficult to form a jet state [157,158].

For the influence of process parameters, with the increase of voltage regulation, the fiber diameter will gradually decrease. If it is too high, it will form bonding and uneven diameter. If the voltage is too low, although the fiber diameter increases, there will be beads. When the injection rate increases, the drying time of the solution decreases and the fiber diameter increases. When the drying rate is low, the drying time becomes longer and thinner fibers can be stretched. Similarly, if the distance between the needle and the collector is too close or too far, beaded fibers will be formed due to improper drying time [159,160]. For the influence of environmental parameters, the increase in temperature will reduce the fiber diameter, thereby increasing the viscosity of the solution. At low humidity, the solvent can be rapidly evaporated and dried [161].

### 2.4. Comparison of Electrospinning Technology with Other Nanofiber Technologies

In addition to electrospinning technology can produce nanofibers, other production methods can also be used (Table 2), such as stretching [162], self-assembly [163], phase separation [164], template synthesis [165], melt spraying [166], freeze-drying [167], solvent casting [168], laser ablation [169], chemical vapor deposition [170], solution blowing [171], carbon dioxide (CO_2_) laser supersonic stretching [172], force spinning [173], dry and wet spinning [174], electrohydrodynamic (EHD) printing [175].

## 3. Herbal Medicine Loaded into Electrospun Nanofibers

**Figure 6:**
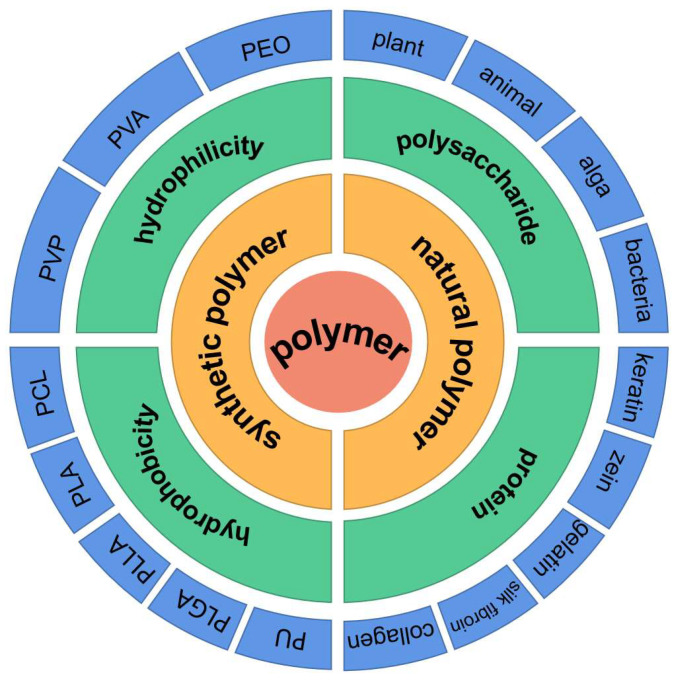
Classification of polymers used in electrospun herbal medicine nanofibers.

Herbal medicine cannot be successfully incorporated into nanofibers because the viscosity of the solution cannot reach the electrospinning process conditions [176]. In this review synthetic and natural polymers that have been tried with commonly used herbal medicines are listed in Figure 6.

### 3.1. Synthesis of Polymer Loaded with Herbal Medicine

Synthetic polymers used in the electrospinning of herbal medicines are generally classified into hydrophobic and hydrophilic classes according to functional applications. They are both biocompatible and biodegradable. The commonly used hydrophobic polymers are polycaprolactone (PCL), polylactic acid (PLA), polyurethane (PU), polylactic acid-glycolic acid copolymer (PLGA) and poly (L-lactic acid) PLLA. In addition, hydrophilic polymers are polyvinyl alcohol (PVA), polyethylene oxide (PEO) and polyvinylpyrrolidone (PVP). In addition to these common synthetic polymers, there are polyesters such as Eudragit E100 (EE100), Carbomer (polyacrylic acid), nylon 66 and polyhydroxyalkanoates (PHA).

#### 3.1.1. Hydrophobicity

Polycaprolactone (PCL) and polylactic acid (PLA) are commonly mixed with herbal medicine for the wound dressing’s application. Poly (ε-caprolactone) (PCL) is a hydrophobic semi-crystalline synthetic polyester. Polylactic acid (PLA) is an aliphatic polyester derived from starch and sugar. Therefore, it is considered as an environmentally friendly alternative to petroleum-based polymers and has many similar characteristics of thermoplastics. Hydrophobic properties of these polymers have given them good mechanical properties and slow dissolution rate, which can promote long-term healing and low inflammatory response [13,177,178,179,180]. However, high hydrophobicity also has its drawbacks because it is not suitable for proliferation and cell adhesion, which limits the effect of wound healing to a certain extent. Therefore, surface hydrophilicity can be improved by blending them with hydrophilic and natural polymers [181,182]. At the same time, the incorporation of herbal medicine extract can effectively promote wound healing and tissue regeneration.

**Figure 7:**
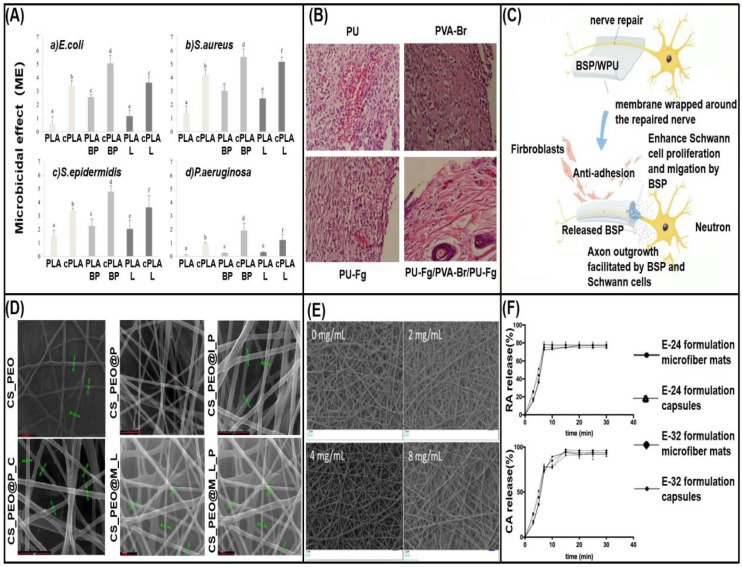
Synthetic polymer-loaded herbal medicine nanofibers. (**A**) Antibacterial activity of fibers against different bacterial strains, reprinted with permission from [49] copyright 2021, Polymers. (**B**) Nanofibers induced skin tissue, reprinted with permission from [20] copyright 2021, Springer Nature. (**C**) Functional schematic of BSP/WPU nanofibers, reprinted with permission from [187] copyright 2020, Elsevier. (**D**) SEM images of CS/PEO nanofibers with different active ingredient combinations (I = insulin, P = propolis, M = Manuka honey, L = L-arginine, C = *Calendula officinalis*), reprinted with permission from [51] copyright 2021, Elsevier. (**E**) SEM images of PVA/PVP fibers with different NFE contents, reprinted with permission from [93] copyright 2021, Foods. (**F**) In vitro release curve of Rosmarinic acid and carvacrol [47], reprinted with permission from [47] copyright 2019, Molecules.

Salami et al. [183] used poly (ε-caprolactone)/poly (vinyl alcohol)/collagen (PCL/PVA/Col) to load the Momordica charantia extract. After incorporation, the wettability of the nanofibers was improved, and the morphology, porosity, water vapor permeability and swelling characteristics were not significantly affected. In vitro results showed that the prepared nanofibers had blood compatibility, cell compatibility, and prevented bacteria from penetrating through the dressing, providing an appropriate environment for wound healing. Milanesi et al. [49] developed a bioactive dressing for chronic wound healing. Polylactic acid (PLA) and black pepper essential oil (BP-EO) were blended in acetone solution, electrospun into nanofibers, and then coated with medium molecular weight chitosan (CS). The results show that the CS coating improves the hydrophilicity of the fiber and forms a biocompatible hydrophilic film for easy treatment. At the same time, it enhances the antibacterial ability of EO alone and promotes cell adhesion and proliferation (Figure 7A).

Due to the excellent properties of PLA, researchers developed a poly (L-lactic acid) (PLLA) polymer. Similar to polylactic acid, it has high mechanical and biodegradable properties, which makes it recognized in bone tissue applications. At the same time, the incorporation of herbal medicine into electrospun PLLA fibers can improve hydrophilicity and osteoinductivity. Parvathi et al. [184] demonstrated that PLLA nanofibers with a high percentage of Cissus quandrangularis (CQ) are hydrophilic and can induce osteogenic differentiation of MSCs. In addition, polylactide-glycolide (PLGA) is also widely used in tissue engineering because of its low cost, clear structure, reliable mechanical properties and degradation kinetics, as well as good biocompatibility and biodegradability. Wang et al. [185] prepared polylactide-glycolide (PLGA)/polyε-caprolactone (PCL)/Zdextran (DEX) composite membranes and loaded with plastrum testudinis extract (PTE), a herb commonly used to treat chronic diseases caused by intervertebral disc degeneration (IDD). The results show that the composite nanofibers have uniform diameter distribution, good mechanical properties, moderate degradation rate and cell compatibility. At the same time, it has anti-inflammatory and promoting cell proliferation effects on annular fibers (AF).

Polyurethane (PU) as a biocompatible hydrophobic polymer is also widely used in medical applications. Due to the porous structure, excellent oxygen permeability, high efficiency of controlling wound moisture, good barrier and mechanical properties, it has been studied in wounds. Since it can absorb exudate from the wound area, control oxygen and water permeability, and accelerate wound healing process [186]. Mir et al. [20] prepared a PU-Fg/PVA-Br/PU-Fg sandwich dressing containing ferula gum (Fg) and bromelain (Br). The fibers have good tensile strength, antibacterial properties against *E. coli*, and dressings that reduce pain area, bleeding, and inflammation (Figure 7B), and can be used for topical administration and treatment of bedsores. At the same time, there are also achievements in nerve tissue engineering. Chen et al. [187] used a kind of waterborne polyurethane (WPU). This electrospun membrane is environmentally friendly, can be incorporated into polar polymers and aqueous dispersions, and exhibits excellent anti-adhesion properties without cytotoxicity. Simultaneously loaded with *Bletilla striata* polysaccharide (BSP), an active ingredient extracted from East Asian plants that is anti-inflammatory and promotes cell proliferation. This composite fiber directly promotes the growth of axons to achieve the purpose of repairing nerves (Figure 7C).

#### 3.1.2. Hydrophilicity

Compared with hydrophobic synthetic polymers, hydrophilic polymers have good film-forming properties, fast dissolution rate and excellent biological adhesion. Due to their versatility, they are used in delivering herbal medicine, skin and wound repair applications and food packaging. Polyvinyl alcohol (PVA) is a water-soluble semi-crystalline petroleum-based polymer, colorless and tasteless. Polyvinylpyrrolidone (PVP) has good biocompatibility and suitable tensile properties. Polyethylene oxide (PEO) is another hydrophilic polymer besides PVA and PVP polymers, which is often used as a drug carrier matrix. They can maintain good moisture content, easy to adhere to the skin wounds, reduce inflammation, accelerate the proliferation of skin cells and wound contraction without scarring [34]. In addition, it can be used as a food active packaging film, and by incorporating natural active ingredients of herbal medicine to improve the quality and safety of packaged food. However, high water solubility also hinders practical applications. In order to overcome this problem, mixtures with other polymers and cross-linking agents can be used to improve the functionality and applicability of the material, allowing for appropriate release rates and concentrations of active compounds [13,188].

In bioactive wound dressings, Jham et al. [189] used polyvinyl alcohol to incorporate natural polymer silk fibroin (PVA-SF), and selected *Elaeagnus angustifolia* (EA) extract as the biological antibacterial active substance of the dressing, which improved the viability of fibroblasts. In order to improve the biocompatibility of PEO and design dressings suitable for chronic wounds, Oana et al. [51] added chitosan CS with extracellular matrix ECM characteristics, which was mixed with different active ingredients (Figure 7D). The CS/PEO nanofibers containing the active ingredient of Propolis-*Calendula officinalis* extract showed improved hemolysis index, non-toxicity, and significant free radical scavenging and antibacterial ability against *Staphylococcus aureus*. Chen et al. [190] prepared natural silk fibroin (SF)/polyvinylpyrrolidone (PVP) nanofibers and loaded (baicalein BAI) to solve swelling in order to develop electrospinning non-woven mats with anti-inflammatory and antibacterial functions. Compared with commercially available Tegaderm dressings, this wound dressing shows high biocompatibility and also promotes wound collagen fibers and angiogenesis. For the preservation of food, in order to improve the stability, Peng et al. [93] made efforts to successfully develop a biocomposite film of polyvinyl alcohol (PVA) and polyvinylpyrrolidone (PVP) encapsulated *Nervilia fordii* (NFE) by electrospinning. The fiber morphology is beadless and smooth (Figure 7E). NFE has high encapsulation efficiency, retains antioxidant capacity, improves thermal stability, and also proves the ability to extend shelf life.

#### 3.1.3. Other Herbal Medicine Polyester

In addition to common synthetic polyesters, some copolymers have also been used in medical applications, such as pH-sensitive polymers, higher thermoplastic polymers, and bacterial-derived synthetic polymers. Eudragit E100 (EE100) and Carbomer are pH-sensitive polymers. Eudragit E100 (EE100) is a cationic copolymer of butyl methacrylate and methyl methacrylate that swells below 5.0. Juste et al. [47] used it as an oral delivery of antibacterial oregano ethanolic extract (OEE), (Figure 7F) is the release curve of the active compound. Carbomer is a highly hydrophilic polymer that exhibits linear swelling and different viscosities at different pH values. Therefore, the use of this polymer in the presence of saliva and mucus can obtain better mucosal adhesion properties. Simzar et al. [21] loaded *Ziziphus jujuba* extract for periodontal treatment. PA66, also known as nylon 66 (polyamide polymer), is a semi-crystalline material with high melting point, while maintaining strength and stiffness at higher temperatures. As a skin scaffold, nylon 66 has the smallest inflammatory response. To better mimic skin elasticity, Simzar et al. [16] incorporated *B.vulgaris* into nylon for skin tissue engineering. Polyhydroxyalkanoate (PHA) is a naturally occurring bio-based polyester that has the characteristics of traditional petroleum-derived thermoplastics and is biodegradable. Nuttapol et al. [22] used as a carrier for loading the traditional Thai herbal extract Plai oil, it can be used as a transdermal patch to relieve pain.

In Table 3, the nanofibers with excellent properties prepared by electrospinning of herbal medicine extracts under synthetic polymer carriers are listed.

### 3.2. Herbal Medicine Loaded Natural Polymer

In the natural polymer of electrospun herbal medicine, the commonly used can be divided into two categories, namely polysaccharide and protein. The polysaccharide from the source, and can be divided into animal polysaccharides, plant polysaccharides, algae polysaccharides and bacteria source polysaccharide. Similarly, protein can be divided into collagen, silk fibroin, corn protein and keratin because of its main components and functions.

#### 3.2.1. Polysaccharides

##### Animal Polysaccharide

Chitosan (CS) and hyaluronic acid (HA) are the animal polysaccharides being researched in preparing herbal medicine nanofibers. CS (β-1,4-glycosidic bond linearly linked glucosamine) is the second most abundant natural cationic polysaccharide on the earth after cellulose. It exists in the shells of crustaceans, insects or fungi such as crabs and shrimps. It is a carbohydrate polymer (glycosaminoglycan) produced by the alkaline heterogeneous deacetylation of natural chitin. The content of N-acetylamino and d-glucosamine depends on the degree of deacetylation, and they are the main components of dermal tissue [205]. CS has been widely used in biomedical applications due to its high absorption, cost-effectiveness, biodegradability, immunostimulatory properties, non-toxicity, antibacterial, anti-inflammatory and remodeling behavior. In particular, in terms of wound healing and skin regeneration, in addition to their non-toxicity and morphological similarity to natural skin ECM, their oxygen permeability, hemostatic ability, scar prevention, biological adhesion and cell affinity enable them to accelerate skin tissue regeneration [206]. Musawi et al. [4] developed honey/chitosan as the most basic antibacterial matrix, and antibacterial substances rich in AuNPs and capsaicin, the active ingredient of medicinal plants, for progressive antibacterial and repair of damaged tissues. Studies have found that capsaicin-containing nanofiber wound dressings exhibit high levels of cell viability and proliferation, as well as the highest wound healing rate. However, due to the high viscosity of pure CS and poor solubility in water, it is difficult to electrospinning, and the mechanical properties of the spun fiber are poor. Therefore, chitosan is blended with polyethylene oxide PEO, polyvinyl alcohol (PVA), polycaprolactone (PCL) and other polymers to improve performance. In this way, the viscosity of the chitosan solution is reduced and its spinnability is improved, thereby obtaining a fiber having an appropriate diameter [27,207].

**Figure 8:**
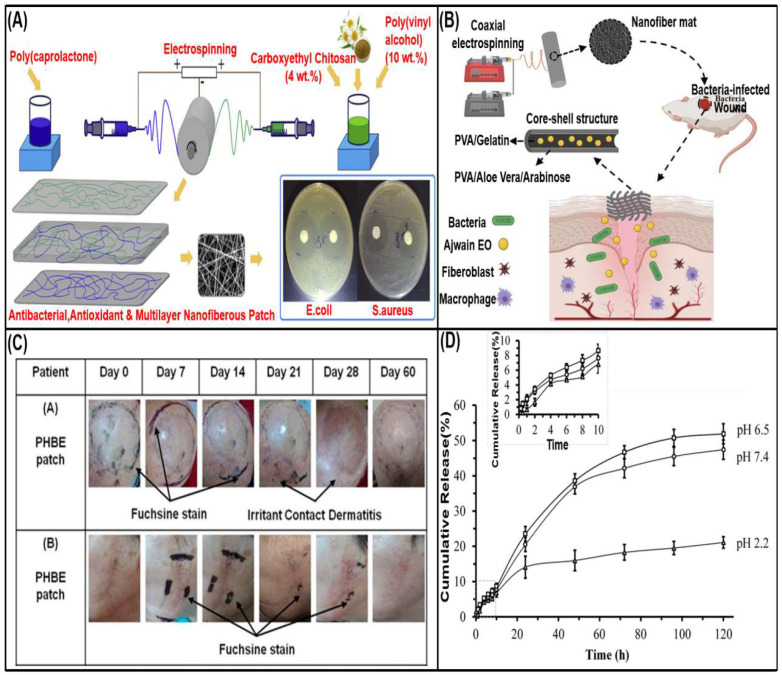
Polysaccharide polymer-loaded herbal medicine nanofibers. (**A**) Electrospun CECS/PVA/chamomile extract nanofibers, reprinted with permission from [209] copyright 2020, Elsevier. (**B**) A schematic diagram of the preparation, structure and use of core-shell nanofiber mats, reprinted with permission from [221] copyright 2021, Elsevier. (**C**) PHBE patch therapy, reprinted with permission from [224] copyright 2021, Cancers. (**D**) X-Ch nanofibers release curcumin, reprinted with permission from [225] copyright 2018, Elsevier.

Hameed et al. [208] encapsulated Clove essential oil in chitosan and polyethylene oxide (PEO) polymers to form NFs, which had no cytotoxicity and showed effective antibacterial and wound healing activities. Mahvash et al. [209] developed a multilayer nanofiber patch, and used water-soluble N-carboxyethyl chitosan (CECS) to avoid the use of organic solvents. PVA was selected to assist the electrospinning of CECS and provide hydrophobic PCL to improve fiber strength. In addition, chamomile extract with antioxidant and antibacterial properties was loaded (Figure 8A). Chitosan (CS) can also be used as a coating of bone implants to promote the orderly formation of bone tissue, while adding herbal extracts as antibacterial ingredients and reducing side effects and toxicity. Kharat et al. [31] used henna and thyme extract loaded into CS/PEO as bone implant antibacterial membrane. In addition to being widely used in medical applications, it can also be used for food packaging. Karami et al. [210] use CH (powdery chitosan) and plant essential oils to protect food from contamination, delay the growth of spoilage microorganisms and lipid/protein oxidation, thereby improving its shelf life and quality, which is specifically mentioned in the application section.

Hyaluronic acid (HA) is a negatively charged linear glycosaminoglycan composed of repeating disaccharide units of 1-4-D-glucuronic acid and 1-3-N-acetyl-D-glucosamine. It is the main component of intracellular, extracellular and pericellular matrices. It is usually present in various parts of the body such as joints, cartilage, and skin tissues. It is responsible for transporting nutrients to cells to help cells degrade products [211]. In electrospinning technology, the insolubility of hyaluronic acid in organic solvents and the high conductivity, viscosity and low volatility of hyaluronic acid aqueous solution hinder the electrospinning process. Snetkov’s research group [212] prepared HA-curcumin electrospun fibers with antibacterial, anti-inflammatory and anti-tumor activities. A metachromatic complex was formed between HA and curcumin in water-organic medium, which stabilized the electrospinning process and improved the fiber properties. They then studied that [213] drug-loaded hyaluronic acid was obtained only in a mixture of water-DMSO solvent. This is because the unstable and hydrophobic curcumin/usnic acid is loaded into a hydrophilic HA matrix to form an electrospinning solution. These studies will lay the foundation for the development of efficient HA wound dressings and new drug delivery scaffolds.

##### Plant Polysaccharide

Plant polysaccharides are also widely used in electrospun herbal medicines, especially cellulose. Pectin and starch are also used as carriers. Cellulose is the main component of green plant cell wall and the most important and abundant natural biopolymer in nature. It is a linear isomorphic homopolymer of β-(1-4)-glycosidic bond-linked D-anhydroglucan with good thermal stability and mechanical stability [214]. The rapid development in the field of nanotechnology has opened up the prospect of cellulose, in the electrospinning process, nanocellulose has a nanoscale size, depending on the dimension (1D, 2D, 3D), its size can be hundreds of nanometers, microns or more [215,216]. Cellulose has several different derivatives, among which cellulose acetate (CA) is the most commonly used in herbal medicine. It is an acetate derivative of cellulose, which is non-toxic, low-cost, easy to prepare, hydrophilic and biocompatible. It can also be easily dissolved in a variety of common organic solvents and become an ideal material for electrospinning [57]. The electrospun nanofibers provide basic advantages such as loadability, controlled release and rich practicality [82]. In addition, its 3D structure and polysaccharides are similar to those of the extracellular matrix (ECM). Therefore, this material has been widely used in the biopharmaceutical processing industry of wound dressings, tissue engineering scaffolds and drug delivery systems, especially in the field of wound dressings to produce different electrospun nanofibers [211]. Ullah et al. [18] developed a bioactive wound dressing that encapsulates *Blumea balsamifera* (BB) oil in CA nanofibers by electrospinning. The fiber loaded with BB oil has biphasic sustained release characteristics, showing good antibacterial effect, cell viability, biocompatibility and air permeability. Although nanofiber CA wound dressing has the characteristics and morphological characteristics suitable for wound dressing application, it lacks appropriate active wound healing ability and antibacterial activity. Farahani et al. [106] prepared a multifunctional cellulose acetate/gelatin/Zataria multiflora-nanoemulsion (CA/Gel/ZM-nano) wound dressing. The presence of protein gelatin in the nanofiber dressing gives it a binding site, which is conducive to cell response, including the adhesion and migration of fibroblasts and keratinocytes to the wound area. At the same time, natural antibacterial plants have appropriate antibacterial activity against *E. coli* and *S. aureus*. In addition, Abbasi et al. [214] incorporated Vent essential oil (OEO) and cellulose acetate (CA) electrospun fibers into gelatin-based films, which can also be used for food active packaging (specifically mentioned in the application).

Gum Arabic (GA) is a branched anionic polysaccharide [217]. On the contrary, bell pectin (BFG) is a non-ionic polysaccharide composed of arabinose, galactose, rhamnose and glucuronic acid. More D-galactose in BFG can provide better water solubility and water holding capacity. In addition, galacturonic acid has high free COO-content and can be used to adjust and modify different properties. At the same time, gum is often used as a gelling agent and adhesive [218]. Carrageenan (CG) is a sulfated linear polysaccharide with the ability to form hydrogels and is functionally modified to sub-connect multifunctional features. However, due to the control of gel properties in the biological environment, poor mechanical stability and high degradation rate are limited, so it is often mixed with other polymers [219]. The mannose/galactose ratio in guar gum determines the synergistic effect and solubility. At the same time, due to the free hydroxyl group and the presence of hydrophobic structure, the interaction with nonionic and ionic drugs can be strengthened. Through this characteristic, surface modification can be carried out to improve stability [220]. These gums/pectins are readily available in nature and have properties such as hygroscopicity, hemostatic properties, water solubility, antibacterial, anti-inflammatory and antioxidant activities. They can be used in tissue engineering, drug delivery, wound dressings, especially in wound dressings. Zare et al. [221] fabricated nanofibrous mats with a core-sheath structure consisting of *Trachyspermum ammi* essential oil/gelatin/polyvinyl alcohol (as the core) and aloe/arabinose/polyvinylpyrrolidone (as the shell), which have long-lasting and strong antibacterial properties and accelerate wound healing of bacterial infections (Figure 8B). Kalachaveedu et al. [220] the electrospinning guar gum/PVA scaffold matrix is rich in four traditional medicinal plant extracts with excellent wound healing, configured to optimize their respective combination ratios, and biocomposite nanofibers have physical and morphological characteristics similar to natural ECM, providing adhesion and proliferation to Gingival Mesenchymal Stem Cells (GMSC), as well as good water absorption and thermal stability. Tensile strength and elastic modulus exceed those of human skin and have been shown to have no dermal toxicity in female rats and to enhance wound contraction and the formation of dermis and epidermis in male rats.

Starch is one of the most widely developed natural polymers in biomedical engineering and biodegradable plastics because of its biocompatibility, biodegradability and low price. It can be obtained from by-products of the agro-food industry, mostly from grains or tubers. Diego et al. [222] prepared an edible film based on waste bananas. However, brittleness and lack of processability are major drawbacks of starch-based products, which are usually improved by mixing with other polymers. Hadisi et al. [223] studied a bioactive gelatin oxidized starch nanofiber for the treatment of secondary burn wounds, containing an antibacterial and anti-inflammatory herbal medicine *Lawononia inermis* extract (Its specific content is shown in the application section.).

##### Algal Polysaccharides

Alginate (Alg) is a natural anionic polysaccharide extracted from brown macroalgae and composed of 1,4-linked D-mannuronic acid and L-guluronic acid units. The immunoregulatory, antioxidant, anticoagulant and anti-tumor effects of seaweed polysaccharides have been widely used in different fields of biomedicine, from drug delivery to regenerative medicine [226]. Researchers have used 3D scaffolds for bone tissue engineering and loaded phenolic purified extracts from *Linum usitatissimum*, demonstrating the potential of bone regeneration [12]. Dressings made from alginate are suitable for skin regeneration and treatment of exudative wounds due to their ability to promote and maintain a physiologically moist environment. Also has excellent biocompatibility, good film-forming properties, easy processing and non-toxic. The Kyritsi team proposed the use of procyanidins and phenolic acids-rich aqueous extract of *Pinus halepensis* bark (PHBE) electrospun polymer micro/nanofibers for the treatment of radioactive dermatitis [224]. The results showed that PHBE patch is a safe preventive treatment of radioactive dermatitis (Figure 8C).

##### Fungal Polysaccharides

In addition to studying the above polysaccharide categories, fungal polysaccharides are sometimes used, most of which are derived from bacteria. Due to their unique properties, they have been discovered and applied by researchers in nanofibers loaded with herbal medicine. For example, dextran is a biocompatible and biodegradable bacterial polysaccharide composed of α-l, 6-glycosidic bonds, in which d-glucopyranose residues are linked to α-1,2-, α-1,3-or α-1,4-side chains. It is easy to obtain and inexpensive, and easy to configure the solution because it is soluble in water and some organic solvents. Luo et al. [227] prepared dextran/corn protein/curcumin hybrid electrospun fibers. When the ratio was 50% dextran and 30% corn protein in acetic acid mixed solution, the fiber membrane showed adjustable wettability and mechanical properties. Encapsulated curcumin showed effective antioxidant activity and controlled release behavior in vitro studies. The results show that the hybrid nanofibers are expected to be used as bioactive delivery systems and edible antibacterial food packaging. In addition, xanthan gum is an extracellular anionic polysaccharide produced by Xanthomonas, containing glucose, mannose, and glucuronic acid. Due to the lateral correlation of the ordered chain sequence, the solution of xanthan gum is weakly gel-like, forming a gel equivalent to the real gel, but may be decomposed under stress conditions. The viscous properties of xanthan gum are determined by hydrogen bonding, which results in high viscosity under low shear stress. Due to its unique physical and chemical properties, it has been used as an encapsulation matrix. Elhamalsadat et al. [225] co-spun xanthan chitosan (X-Ch) and curcumin in formic acid to produce nanofibers. The release of curcumin at pH 2.2 was lower than that in neutral medium, which proved that X-Ch nanofibers could be used as carriers for encapsulating hydrophobic and pH-stimulated release bioactive compounds (Figure 8D).

#### 3.2.2. Proteins

**Figure 9:**
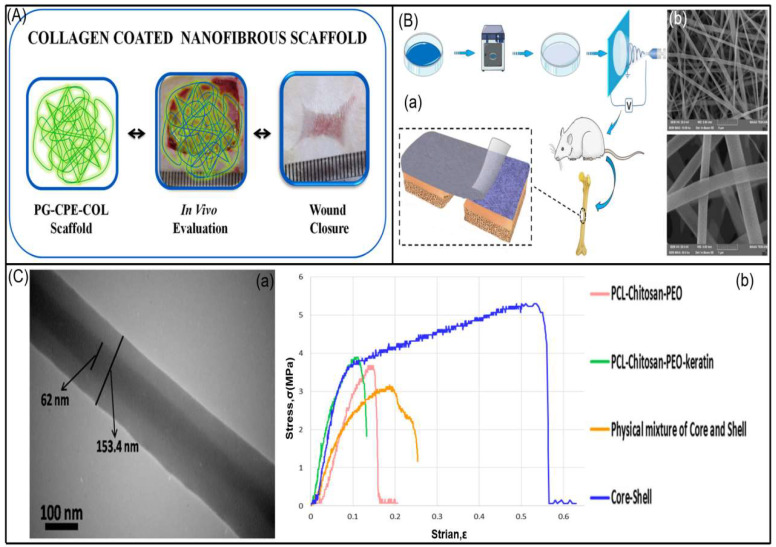
Protein polymer-loaded herbal medicine nanofibers. (**A**) PG-CPE-COL nanofiber scaffold, reprinted with permission from [228] copyright 2017, Elsevier. (**B**) (**a**) Schematic diagram of preparation and implantation method of BER @ PCL/PVP-MC/CS bilayer membrane; (**b**) Diameter distribution under SEM images, reprinted with permission from [229] copyright 2021, Frontiers in Bioengineering and Biotechnology. (**C**) (**a**) Transmission electron microscope (TEM) images of core-shell nanofibers prepared by coaxial electrospinning; (**b**) physical and mechanical properties of electrospun nanofibers, reprinted with permission from [206] copyright 2019, Marine Drugs.

Protein is an indispensable amino acid component in the human body. This kind of polymerization is similar to human ECM and has high biocompatibility. At the same time, loading bioactive substances extracted from herbal medicine can better promote cell adhesion and proliferation, and is widely used in medical applications. Collagen formation mainly constitutes 30% of the body weight of animals. It is the most important protein in the body and the main fibrin in the ECM, supporting the structure of tissues and organs and regulating cell function. Collagen has ductility, bioabsorbability, non-toxicity, antimicrobial properties, high biocompatibility, and can be combined with other copolymers. It is a suitable biological material for nanofibers. However, collagen also has some disadvantages, including difficulties in work, high purification costs, and the risk of disease transmission [211]. Electrospun collagen nanofibers enable ECM to provide additional support for keratinocytes and fibroblasts, allowing them to adhere firmly to the fibers, migrate on the wound bed, regenerate and repair the injured tissue, thereby healing faster. Ramanathan et al. [228] coated deep-sea fish collagen (COL) on Poly (3-hydroxybutyric acid)/gelatin composite nanofiber scaffolds containing antibacterial and antioxidant *Coccinia grandis* leaf bioactive extracts (Figure 9A). This collagen-coated nanofiber scaffold exhibits elevated levels of hydroxyproline, hexosamine, and uronic acid, enhanced growth factor expression, reduced inflammation, and increased wound epithelial regeneration, thereby accelerating healing efficiency. Collagen is also used in bone tissue engineering. In order to ensure bone regeneration and remodeling, Zhang et al. [229] electrospun polycaprolactone (PCL)/polyvinylpyrrolidone (PVP) nanofibers loaded with berberine (BER) to mineralized collagen (MC)/chitosan (CS) cast film to form a double-layer film. Cast film technology overcomes the insolubility of MC and provides feasibility for bone regeneration (Figure 9B). In addition, mineralized collagen, whose fibrous chemical composition and microstructure are similar to natural bone, can provide a more biomimetic microenvironment, and combined with the bioactive component BER, enhanced the proliferation and adhesion of MC3T3-E1 cells and induced bone regeneration at 4 and 8 weeks after implantation of adult rat femoral defects.

Gelatin is the product of acidic or alkaline hydrolysis of animal collagen. It is also the most abundant component in bone and skin. Its composition and biological characteristics are almost the same as collagen. This makes gelatin physically and chemically very similar to the extracellular matrix ECM [33]. It has excellent biodegradability, biocompatibility and non-immunogenicity, and the nanofiber membrane produced by electrospinning technology has the advantages of small pore size, high surface area and physical stability. Gelatin can also be easily cross-linked because of its intrinsic protein structure and a large number of different functional groups and used as a targeted drug delivery carrier [179]. Although gelatin has good functional properties, gelatin has poor mechanical strength, degradability and water resistance [214]. Therefore, mixing PCL with gelatin can produce a scaffold with high mechanical strength and a cell-specific motif suitable for accelerating wound healing [230]. It is sometimes easy to roll up after the film is dried, and the additional base layer can provide structural support for the gelatin nanofibers [231].

In addition, gelatin contains an amino acid sequence similar to Arg-Gly-Asp (RGD), which can promote cell adhesion and migration. Its unique function makes gelatin nanofibers an ideal material for wound dressings. Yao et al. [232] chose a mixed gelatin made from collagen from freshwater fish skin and pork collagen. Fish gelatin can reduce costs and some infectious diseases on the one hand, and has relatively high thermal stability on the other hand. They designed double-layered scaffolds by electrospinning PVA/gelatin nanofibers containing the root of *Lithospermum erythrorhizon* (LR) extract onto chitosan scaffolds modified by glutaraldehyde vapor. The Shikonin component in the extract has many pharmacological properties such as antibacterial, anti-inflammatory and promoting cell transformation. It is a traditional herbal medicine used to treat skin wounds. The porous structure of the scaffold can absorb the exudates well and exhibit greater cell adhesion. LR extract provided the highest wound recovery rate in vivo. At the same time, gelatin can also form a viscous matrix structure, making it an excellent candidate for food bio-packaging. Hani et al. [30] encapsulated the crude ethanol extract of *Moringa oleifera* Lam. (MO) in the biopolymer matrix of fish gelatin by electrospinning. The encapsulation efficiency was more than 80%, and the content of total phenols and flavonoids and antioxidant capacity were hardly affected.

Silk fibroin (SF) is a natural structural protein, commonly extracted from silk worms, and is one of the strongest and toughest biomaterials. It is a major protein component composed of a heavy (H) chain and a light (L) chain, which is connected by a disulfide bond at the C-terminus [233]. The adaptability and simple low-cost production of silk fibroin make it an ideal candidate for biomedical applications. At the same time, it has unique biocompatibility and biodegradability, low immunogenicity, anti-inflammatory, permeability, good cell adhesion and ability to control the release of growth factors [234]. Compared with commonly used biopolymers such as collagen and gelatin, SF also exhibits acceptable mechanical properties and controllable biodegradation rate [15,189]. Ma et al. [60] loaded Cirsium Japonicum DC extract with obvious hemostatic, anti-inflammatory and analgesic effects into SF nanofiber matrix. The results showed that the incorporation of this Chinese herbal extract significantly improved the hemostatic properties of SF nanofiber matrix.

Zein is one of the hydrophobic plant proteins (the main storage proteins of maize) and is mainly composed of amino acids such as leucine (20%), glutamic acid (21–26%), alanine (10%) and proline (10%). Corn protein has good biodegradability, biocompatibility, flexibility and high microbial resistance, non-toxic and antioxidant. Rad et al. [217] developed PCL/Zein/GA nanocomposite scaffolds containing *Calendula officinalis* extract as a potential matrix for skin regeneration. *Calendula officinalis* has many ingredients and a variety of effects, such as blood clotting, anti-bacterial anti-inflammatory, free radical inhibition. Through suspension and double nozzle electrospinning, the results clearly showed controlled release, biocompatibility, antibacterial properties and cell growth. In addition, it has excellent film-forming and gas barrier properties, making it an ideal matrix for edible active food packaging applications [235].

Keratins are structural elements in many organisms. The intermolecular bonding of disulfide cysteine and the existence of intermolecular and intramolecular bonds of various amino acids make keratin a highly stable protein with unique physicochemical properties. Zahedi et al. [206] used coaxial electrospinning technology, aloe extract/PEO as the core, PCL/chitosan/keratin nanofibers as the shell. Due to the protection of the shell, the herbs are allowed to be incorporated into the membrane. *Aloe vera* gel is composed of 99% water. This therapeutic herb has excellent antibacterial properties. The presence of keratin improves adhesion and slightly increases tensile strength (Figure 9C).

In Table 4, nanofibers with excellent properties prepared by electrospinning of herbal medicine extracts with natural polymer carriers are listed.

## 4. Application of Herbal Medicine Loaded Nanofibers

### 4.1. Drug Delivery System

**Figure 10:**
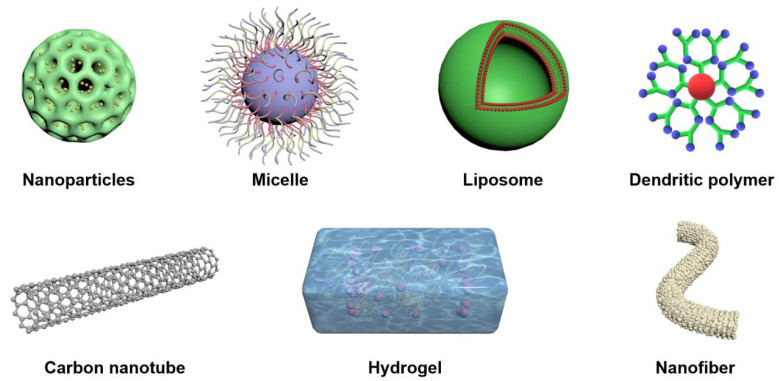
Herbal nanomedicine system.

Herbal nanomedicine (HNM) delivery systems have become an attractive research topic in recent years because of their precise drug delivery, reduced toxicity and enhanced activity, and the potential to overcome herb-related problems. Therefore, many drug delivery systems are used to improve bioavailability. For example, polymer nanoparticles [239], hydrogels [12,42], micelles [240], nanotubes [241], dendrimers [242], liposomes [243], and nanofibers [244] (Figure 10). Bioactive molecules are incorporated into appropriate nanocarriers to improve their bioavailability and effectiveness by reducing toxicity and sustained drug release at target sites, and enhance their physicochemical stability and solubility.

These drug delivery systems have varying degrees of disadvantages, such as difficulty in controlling the proportion of drugs in liposomes and the preparation of emulsions [55]. Compared to other drug delivery systems, electrospinning is a “one-step” process using cheaper raw materials. Electrospun nanofibers are used as drug carriers in drug delivery systems due to their high functional properties and different controlled drug release curves (such as immediate [245], sustained [246] and biphasic release [247]). Encapsulating herbal medicine into nanofibers has several advantages: (1) especially in the oral form to improve patient compliance, allowing self-management [248], and allowing local administration, greatly reducing the toxicity of systemic drugs [59]; (2) The therapeutic effect can also be maximized by increasing bioavailability and maintaining drug concentration [197]; (3) Increases drug solubility and promotes rapid drug release [47].

**Figure 11:**
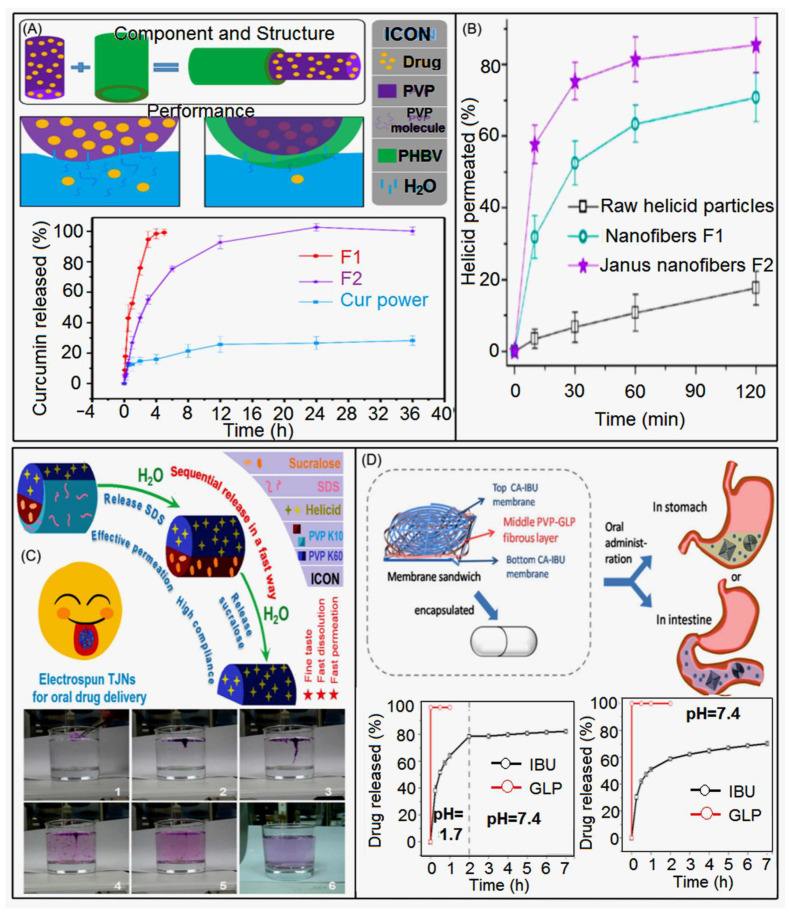
Nanofiber herbal medicine delivery system. (**A**) Drug release mechanism (**the up section**) and curcumin release curve of core-sheath structure nanofibers (**the down section**), reprinted with permission from [136] copyright 2022, Polymers. (**B**) Membrane permeability of Helicid, reprinted with permission from [63] copyright 2018, American Chemical Society. (**C**) Release mechanism of three-fluid hydrophilic Janus structure and rapid disintegration experiment of fiber membrane, reprinted with permission from [204] copyright 2022, Springer Nature; (**D**) Hydrodynamic EHD printing combined with electrospinning technology, the dissolution and release mechanism of fiber membrane in gastric juice and intestinal juice, reprinted with permission from [175] copyright 2018, American Chemical Society.

Liu et al.’s research group [136] used a combination strategy of hydrophilic PVP and hydrophobic poly (3-hydroxybutyrate-co-3-hydroxyvalerate) PHBV to prepare coaxial fibers. The hydrophobic PHBV polymer was used as a blank protective layer to improve the drug release characteristics of curcumin (Cur) with poor water solubility. Compared with the rapid dissolution and release of pure PVP, the hydrophobic layer was added, and the water molecules slowly penetrated the PHBV fiber layer. The inner PVP and Cur were slowly released into the medium (Figure 11A) to improve its solubility and improve the difficulty of curcumin absorption. At the same time, the drug-loaded fiber of the core-sheath structure has no burst release phenomenon, and the Cur release time can be extended by more than 24 h. This continuous administration method significantly improves the therapeutic utilization efficiency of Cur with poor water solubility. Wang et al.’s team [63] designed a hydrophilic Janus structure nanocomposite with PVP K10/sodium dodecyl sulfate SDS transmembrane enhancer and PVP K90/Helicid on both sides for rapid dissolution and transmembrane permeation of oral poor water-soluble Helicid. Although there was no significant difference in their in vitro dissolution release compared to single-spun drug-loaded fibers. However, Janus nanofibers have better hydrophilicity and better rapid dissolution performance. Tongue mucosal penetration is also an important consideration. The permeability of Janus nanofibers can reach 16.4 times higher than that of traditional oral Helicid by more than 10 times (Figure 11B). This is because the transmembrane enhancer SDS plays a crucial role and improves its performance. This method can give insoluble drugs faster and play a role in rapid treatment.

Based on the research of Wang et al., Yu’s research group [204] optimized the design of a new three-stage hybrid nanofiber (TJNs) composed of three composite materials: Helicid-polyvinylpyrrolidone (PVP K60)/sodium dodecyl sulfate (SDS)-PVP K60/sucralose-PVP K10. Two disintegration experiments (with one drop of water and one glass of water) and in vitro dissolution tests (Figure 11C) showed that TJNs had rapid dissolution properties, and that loaded SDS and sucralose could be released in a certain order before Helicid, increasing the convenience of drug delivery, while the addition of sweeteners was friendly to the oral delivery system because it increased patient compliance. Wu et al. [175] combined electrohydrodynamic EHD printing with electrospinning to prepare a fiber membrane with a flexible sandwich structure that combines Chinese and Western medicines and is finally assembled into an oral capsule (Figure 11D). The printed membrane with round top layer and square bottom layer is composed of ibuprofen (IBU)/cellulose acetate CA, and the sandwich layer is composed of *Ganoderma lucidum* polysaccharide (GLP)/polyvinylpyrrolidone PVP. In vitro gastric and intestinal fluid simulation tests showed that with the dissolution of the capsule shell, GLP was rapidly and completely released within 1 min, while IBU showed two release forms and released in a two-phase mode. In the initial stage, 60% of IBU was released in a short period of time within one hour, and then it was continuously released for a long time. This design method first selects a capsule form that is easy to carry and swallow on the protective shell, followed by printing to customize the required shape to achieve accurate drug dose, and the final sandwich structure can provide controllable and separate release, which provides a feasible solution for personalized treatment for the oral system.

### 4.2. Wound Dressings

Wound dressings should have skin-like structure, bioactivity and biodegradability [249,250]. The general requirements for an ideal wound dressing are high swelling capacity, porosity, good water vapor permeability, maintaining a humid environment at the wound site, absorbing excess exudate from the wound, essentially non-adherent, easy to handle, non-toxic and non-allergic, providing moderate debridement, providing mechanical stability, and providing protection against microbial invasion and contamination [25,211]. At present, modern dressings are focused on fibers, films, gels, etc. by using natural or synthetic polymers or a combination of both [251]. The electrospun nanofiber membrane is advanced because it has many unique advantageous properties that meet the needs of wound healing [78]. The ultra-fine size of the electrospun fibers ensures mechanical flexibility, thereby ensuring excellent compliance between the nonwoven pad and the wound site. In this way, nanofiber bandages can completely cover the injured tissue, preventing infection and dehydration, as well as heat insulation. On the other hand, the high porosity of the electrospun mesh is conducive to gas permeation, nutrient transport, water retention and exudate absorption. In addition, the morphological similarity and high surface volume ratio of nanofibers to ECM are conducive to cell adhesion, proliferation and differentiation during tissue regeneration. Nanofibers can also be used as carriers to deliver therapeutic substances (such as antioxidants, anti-inflammatory agents and antibacterial agents) directly to the wound site [250,252].

With the improper use of antibiotics and biofilm formation and harmful bacteria continue to mutate, resulting in resistance, which delays the healing period and lead to increased infection, the possibility of other complications [253]. In order to prevent adverse reactions and further enhance the healing ability of wound dressings, plant extracts or herbal medicines with antimicrobial effects can be incorporated into wound dressings to enhance cell regeneration and protect the microbial community in the wound bed from natural wound healing [221]. At present, many medicinal plants are widely used for wound treatment in different parts of the world, especially in Southeast Asian countries. Plant chemicals, such as phenols, tannins, terpenoids, alkaloids and flavonoids, have antibacterial effects [35]. Natural plant antibacterial nanofiber mat as a new research direction, the advantage is that it can inhibit or remove the antioxidant properties of reactive oxygen species, less toxic side effects, environmentally sustainable, easy to obtain and lower cost [252]. The incorporation of herbal medicines and their bioactive extracts can both heal chronic and acute wounds and provide other health care benefits [25].

With regard to medicinal plants, about 25%consist of herbs, trees, shrubs or herbs, and various scientific studies have reported positive effects on various stages of healing [253]. Commonly studied plants for wound healing include *Aloe vera*, Curcuma longa L, Honey, Calendula officinali, *Centella asiatica*, *Azadirachta indica*, Halepensis, *Lawsonia inermis* for burns and *Lithospermum erythrorhizon* for diabetes, as well as Clove and *Nigella sativa* for essential oils (See Table 5).

In addition to these well-studied herbal medicines, it has been reported that Isatis root, a traditional Chinese herbal medicine, is used to treat skin inflammation [120], *Mikania micrantha* [28] with a variety of lactone-active nutrients, Falcaria vulgaris, commonly consumed as a vegetable [29], *Opuntia cochenillifera* rich in minerals and Cacti with high ion absorption [236], *Cirsium japonicum DC* (CJDC) with excellent hemostatic activity [60], *Acanthus ebracteatus Vahl*. [82], *Morinda citrifolia* with nutrients and high biological activity [34], and *Althea officinalis* with mucus-assisted cell properties [37], *Agrimonia eupatoria L*. [23], *Plantago australis Lam*. [32], *Thespesia populnea* [81], *Coccinia grandis* [228], beet [16], *Gymnema sylvestre* [230], baicalein [190], *Matricaria recutita* L. [209], Pulsatilla for the treatment of diabetes [254], *Hypericum perforatum* [255], Peppermint [179] and *Cabreuva* extracted as essential oils [80], *Blumea balsamifera* [18], *Plai* [22], *S. mutica/O. decumbens* [10], Thyme [26]. Since their various pharmacological activities are used in wound healing therapy, natural herbal extracts are becoming a research interest in the field of wound healing.

**Figure 12:**
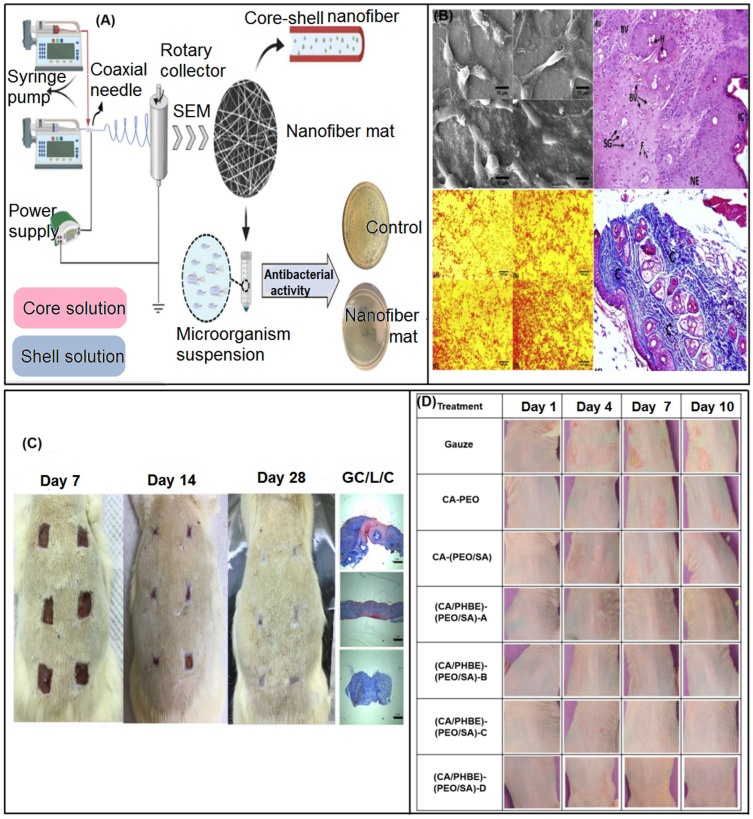
Application of herbal medicine incorporated nanofibers in different wound types. (**A**) CS/PVA/EO-PVP/MD core-shell nanofiber scaffold for wound dressing strategy, reprinted with permission from [10] copyright 2021, Elsevier. (**B**) Effects of the bioactive substance *Lawononia inermis* fiber for burn strategy on cells and collagen and in vivo evaluation (H: hair follicles; bV: blood vessels; f: fibroblasts; c: Collagen; iC: inflammatory cells; sG: sebaceous gland; nE: new epithelialization), reprinted with permission from [223] copyright 2018, Elsevier. (**C**) Treatment of skin regeneration in mice with double herbal medicines for diabetic wounds, reprinted with permission from [231] copyright 2019, Polymers. (**D**) Clinical evaluation of skin inflammation, reprinted with permission from [44] copyright 2019, Materials.

Barzegar et al. [10] studied the core-shell nanofiber scaffold with chitosan (CS)/polyvinyl alcohol (PVA) as the core and polyvinylpyrrolidone (PVP)/maltodextrin (MD) as the shell. *Satureja mutica* (*S. mutica*) or *Oliveria decumbens* (*O. decumbens*) essential oil (EO) is encapsulated in the core of the bracket. The CS/PVA/EO-PVP/MD core-shell scaffold has a uniform bead-free structure and shows appropriate mechanical properties. The addition of EOs enhanced the antioxidant activity of scaffolds (64.12 ± 5.55 and 61.74 ± 3.20%). The final antibacterial test showed that 10% *S. mutica* or *O. decumbens* EO loaded had the best antibacterial effect, expanding the antimicrobial activity of the scaffold. (Figure 12A). Hadisi et al. [223] developed a gelatin/oxidized starch crosslinked nanofiber containing *Lawononia inermis* for the treatment of burn wounds (Figure 12B). The bioactive concentration in nanofibers exceeds 30%, and beads appear due to the decrease of viscosity. In vitro experiments showed that it had good antibacterial properties and could promote fibroblast proliferation and collagen secretion. In the in vivo mouse model, the inflammatory area and wound healing time were reduced. After 4 days of skin regeneration, epithelization, blood vessels, hair follicles, sebaceous glands and well-organized collagen were formed in the correct order and time period. This provides a feasible idea for burn wound treatment.

For the treatment of chronic wounds caused by diabetes, Bo et al. [231] proposed a method of loading two traditional herbal medicines curcumin and Lithospermi radix into gelatin and electrospinning them on chitosan scaffolds to prepare double-layer nanofiber scaffolds (Figure 12C). In the STZ diabetic rat model, the recovery rate was increased by 58 ± 7% on day 7, and the wound produced higher collagen levels and higher levels of transforming growth factor-β (TGF-β) release than other wound dressing-treated wounds. Dermatitis in the skin is the most common, especially inflammation caused by allergies and sunburns caused by ultraviolet light. Kotroni et al. [44] studied alginate micro/nanofiber dressings containing anti-inflammatory activity *Pinus halepensis* bark (PHBE) (Figure 12D). The study found that the patch with a concentration of 26.2% *w*/*w* PHBE was the highest concentration of electrospinning and showed the best anti-inflammatory activity. In the back skin radiation test of SKH-1 female hairless mice, there were obvious erythema and damage. The use of this dressing can reduce inflammation and shorten the healing period. At the same time, the proportion of the degree, range and depth decreased, so this skin anti-inflammatory patch may be an ideal candidate.

### 4.3. Tissue Engineering

Due to tissue and organ failure caused by injury, disease, congenital disability and aging or any other event, previous transplant surgery will have the risk of immune rejection and surgical failure. However, with the advancement of nanotechnology, tissue engineering scaffolds have become a promising method, which can be used as drug carriers for effective target delivery and simulated extracellular matrix implantable biomaterials to repair or replace damaged tissues, thereby improving the health and quality of life of patients [256,257].

The ideal scaffold for tissue engineering should have three main characteristics. (i) provide biocompatibility; (ii) reliable mechanical; (iii) Similar to extracellular matrix (ECM) in composition and structure [258]. Electrospinning fibers with high porosity, high permeability, and high surface area can improve nutrient exchange, metabolism, and control cell signaling, mimic ECM, and enhance cell adhesion, proliferation, migration, and differentiation [192]. Biodegradable materials are often used to simulate the physiological environment to enhance biological non-immunogenicity. Due to the flexibility of material selection and the ability to adjust scaffold performance, electrospinning scaffolds have been used in a range of tissue applications, including blood vessels, bones, and nerves. In practical research, we will add herbal medicines and their extracts to meet the nutritional and biological needs of specific tissue-forming cells and enhance the repair and regeneration of damaged tissue/organ targets [2].

For example, in bone applications, the commonly used herbal medicines are Epimedium and *Cissus quadrangularis* (CQ) because of their broad pharmacological effects. Icariin (ICA) is the main component of Epimedium, and its active ingredient is total flavonoids. It has angiogenesis, anti-osteoporosis, anti-inflammatory and induce bone regeneration, increase the synthesis of bone extracellular matrix [234]. *Cissus quadrangularis* (CQ) is widely used in India. It is usually extracted by ethanol distillation and has antibacterial and antioxidant activities. Due to its high calcium and phosphorus content, CQ stimulates metabolism and increases the absorption of minerals. Therefore, it promotes the proliferation and osteogenic differentiation of bone marrow-derived MSCs (mesenchymal stem cells) [195]. Other researchers are also looking for new solutions from more herbal medicines, such as Plastrum Testudinis [185] for the treatment of disc inflammation, *Elaeagnus angustifolia* (EA) [52] for the treatment of rheumatoid arthritis and osteoarthritis, Clove [109] and *Ziziphus jujuba* [21] for the treatment of periodontal disease, and Amla oil [56] with rich antioxidant and mineral components. Berberine [229] for increased membrane permeability, *Spinacia oleracea* (SO) [19] for high quercetin content, Henna/Thyme [31] and cinnamon [43], Naringin [259] for antibacterial implants.

**Figure 13:**
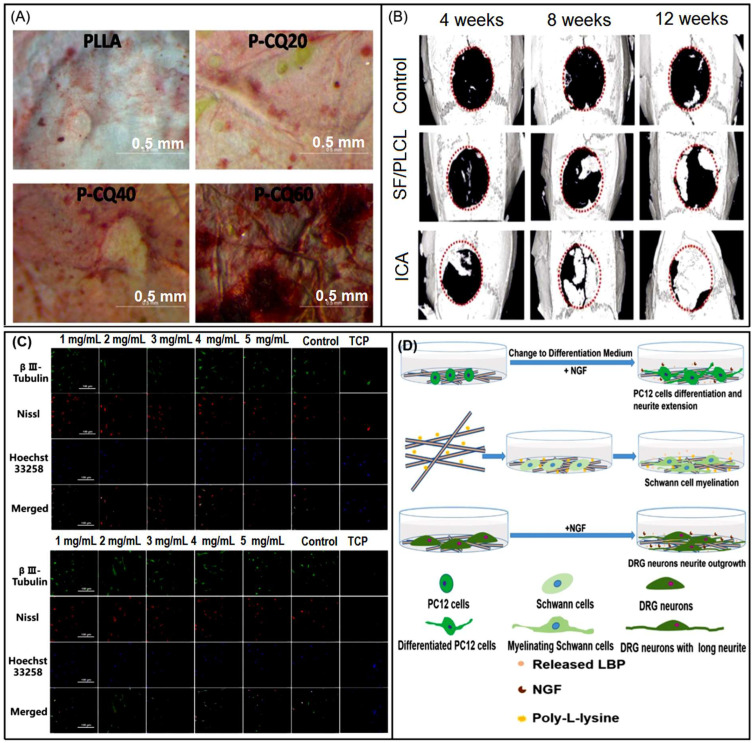
Application of pharmacological activity of herbal medicine in tissue engineering. (**A**) Mineralization micrograph, reprinted with permission from [184] copyright 2017, Elsevier. (**B**) Potential of ICA-SF/PLCL scaffold for bone regeneration, reprinted with permission from [234] copyright 2017, Scientific Reports. (**C**) The effect of (PLGA/MWCNTs/SF)-Catapol scaffold on neuronal differentiation of hASCs; (**a**) Three days; (**b**) 5 days [15], reprinted with permission from [15] copyright 2015, Biomedical Engineering Society. (**D**) Effect of PLGA-LBP scaffold on proliferation and differentiation of PC12 cells and myelination of Schwann cells to promote nerve growth, reprinted with permission from [50] copyright 2018, Scientific Reports.

Parvathi et al. [184] prepared a PLLA nanofiber rich in high percentage of CQ (20% and 40%, respectively) by electrospinning. The loading of herbs makes the film hydrophilic and significantly enhances the adhesion, proliferation and osteogenic differentiation of mesenchymal stem cells (mainly on P-CQ20 and P-CQ40) without providing other osteogenic supplements. The results of the final mineralization study (Figure 13A) showed that the mineralization process was accelerated during the 14-day simulation. Therefore, the electrospun nanofibers loaded with CQ reflected the osteoinductive ability and could be used as a potential candidate material for bone tissue engineering applications. Yi et al. [234] incorporated icariin into silk fibroin/poly L-lactide-caprolactone nanofiber membrane (ICA-SF/PLCL) as an osteoinductive factor by coaxial electrospinning. The prepared nanofibers, ICA, continued to be released in a continuously controllable manner after initial rapid release. In vitro experiments showed that the membrane had good biocompatibility with mesenchymal stem cells (BMMSCs) and significantly promoted its osteogenic activity. After 12 weeks of implantation in vivo, new bone tissue has been formed in most defect areas, and the volume and density can reach 15.95 ± 3.58 mm^3^ and 14.02 ± 0.93% (Figure 13B). All these prove the hope of ICA-SF/PLCL nanofiber membrane in guided bone regeneration (GBR).

In addition to the challenge of bone tissue repair, nerve injury caused by certain external factors is also a difficult problem in tissue engineering, because neurons are fragile, weak regeneration ability and largely no longer formed. This requires timely and effective treatment before causing cell death. Electrospinning incorporating herbal medicines to promote nerve regeneration may be a promising direction. Catalpol is an iridium glucoside isolated from the Chinese herb Coptidis Rhizoma and is widely used to treat stroke and aging diseases due to its neuroprotective effects.Guo et al. [15] added it to the poly (lactic-co-glycolic acid) PLGA/multi-walled carbon nanotubes MWCNTs/silk fibroin SF nanofiber scaffold. The results showed that Catalpol significantly up-regulated the expression of βIII-tubulin and Nissl, and induced hASCs to differentiate into neuron-like cells (Figure 13C), which may provide new ways and potential for nerve regeneration.It has been reported that *Lycium barbarum* polysaccharide (LBP) in edible *Lycium barbarum* has also been proved to have the possibility of neuroprotection and nerve recovery. Wang et al. [50] electrospun PLGA-LBP core-shell nanofiber scaffolds in a coaxial fashion. From the results of cell proliferation, the released LBP significantly enhanced NGF-induced PC12 cell proliferation and neuronal differentiation. In addition, Schwann cells were myelinated to promote neurite growth of DRG neurons (Figure 13D). Therefore, LBP has a positive effect on the proliferation and differentiation of nerve cells in vitro. It can be used as a neuroprotective drug encapsulated in electrospun nanofibers and may be a potential candidate for tissue engineering scaffolds for peripheral nerve regeneration.

### 4.4. Food Packaging

With the improvement of quality of life, people pay more and more attention to food safety, and consumers begin to worry about the side effects of synthetic food additives and plastic packaging on human health. Therefore, the development of bio-based packaging for food preservation has always been a hot topic. In this field, active nanofiber membranes are manufactured by combining antibacterial and antioxidant compounds with biopolymer materials to reduce environmental problems caused by microbial contamination and non-degradable materials, prolong the shelf life of food, and meet the needs of health and environmental protection [11].

Among the substances with antibacterial properties, some secondary metabolites of herbaceous plants, such as essential oils (EO), are mainly terpenoids, which have antioxidant and antibacterial properties. For example, *Ziziphora clinopodioides* essential oil (ZEO) and sesame essential oil (SO) [210], *Laurus nobilis* and *Rosmarinus officinalis* essential oils [235], Cinnamon essential oil [260], *Oliveria decumbens* Vent essential oil (OEO) [214] and other Chinese herbal bioactive substances with antimicrobial effects such as sucrose seed meal powder [13], Pomegranate peel [237], *Acalypha indica* [193], *Nervilia fordii* [93] for the preservation of high-fat foods and *Butia catarinensis* [261] for the outer packaging of biological foods. However, the effective active ingredients are usually affected by poor solubility, high volatility, and environmental factors (heat sensitivity) [188]. In addition, EO has a certain irritating odor, which may affect the original taste of the food, so the dosage can be used in a small amount, which will limit the function in practical application [235]. Electrospinning technology does not require high temperature, and the active components will not be decomposed to a large extent. The final spun active nanofibers have high encapsulation efficiency to improve solubility and can be continuously and effectively released [210].

**Figure 14:**
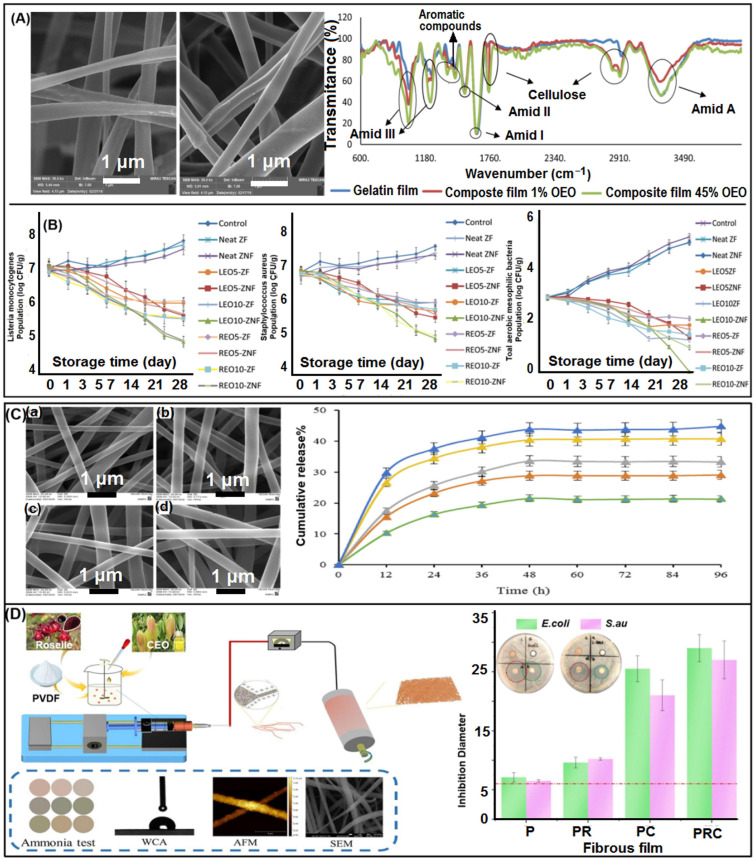
Food packaging application of herbal medicine extracted as essential oil. (**A**) Fiber morphology and FTIR analysis of gelatin/essential oil film, reprinted with permission from [214] copyright 2021, Springer Nature. (**B**) Antibacterial experiment, reprinted with permission from [235] copyright 2020, Elsevier. (**C**) Fiber morphology and drug release curve incorporated with essential oils, (a) and (c) are loaded with sesame seed oil, and (b) and (d) are loaded with *Z. clinopodioides* essential oil [210], reprinted with permission from [210] copyright 2021, Elsevier. (**D**) Active intelligent packaging, reprinted with permission from [260] copyright 2022, Elsevier.

Abbasi et al. [214] encapsulated *Oliveria decumbens* Vent essential oil in cellulose acetate (CA) for electrospun fibers and composited it with gelatin to form a film. This composite has better tensile strength and lower tensile rate than pure gelatin, and because CA is hydrophobic, the film obtains better water resistance and can better inhibit bacterial reproduction (Figure 14A). Goksen et al. [235] used *Laurus nobilis* and *Rosmarinus officinalis* essential oils to extract essential oils to study the antibacterial properties of coated cheese. They have an effective inhibitory effect on *Listeria monocytogenes*, *S. aureus* and mesophilic bacteria, and LEO was found to be more antibacterial than REO by reducing the number of bacteria and the effective duration (Figure 14B).

Karami et al. [210] added ZEO/SO to chitosan (CH)/flaxseed mucilage (FM), the prepared fiber had a larger diameter due to the decrease of viscosity and conductivity, but the overall morphology was good, no agglomeration and phase separation, and could be continuously released within 96 h to achieve long-term antibacterial effect (Figure 14C). Zhang et al. [260] proposed an interesting research idea, and prepared a multi-functional electrospinning PVDF/CEO/RS fiber membrane by electrospinning. Due to the presence of Roselle anthocyanin, the prepared film has a sensitive color reaction, so it can be used to detect the freshness of meat. Under the action of essential oil, it has good antibacterial activity against *E. coli* and *S. aureus* and extends the shelf life (Figure 14D), which provides great potential for active and intelligent packaging.

## 5. Conclusions and Outlook

Herbal medicine is used worldwide and has a variety of effective active ingredients (such as flavonoids, tannins and terpenoids), which can be used for antibacterial, anti-inflammatory and enhancing cell proliferation. To develop a novel herbal medicine delivery system, nanofibers are used to deliver herbal medicine extracts and natural bioactive components due to their high surface area and porosity, as well as the characteristics of simulated ECM. Electrospinning technology has been applied to various medical fields, such as drug delivery, wound dressings, tissue engineering scaffolds and biological food packaging. Simultaneous synthesis of polymers with natural polymers and mixed polymers can optimize the biocompatibility and biodegradability of nanomaterials, improve physical and chemical properties, and enhance the functionality of nanofibers.

At present, development of electrospun nanofibers containing herbal crude extracts is in its infant stage. From the application summary of herbal medicine wound dressing, tissue scaffold and active packaging reviewed in this paper, it is feasible to prepare electrospun nanofibers by mixing herbal medicine and polymer. In future, the following directions deserve more attention:

- More research on their safety and effectiveness, especially in clinical practice [262,263,264,265,266];
- The co-encapsulation of novel pharmaceutical excipients such as chiral hydrogels [267,268,269,270], new polymers [271,272,273], and even inorganic materials [274,275,276,277];
- The synergistic actions of electrospun herbal medicines with other chemical active ingredients;
- The applications of electrospun complex nanostructures for manipulating the drug release behaviors of herbal medicines;
- The productions of electrospun herbal medicines on an industrial scale.

## Figures and Tables

**Table 1:** Examples of traditional herbal medicine: components, effects and undesirable properties.

Traditional Herbal Medicine	Component	Effect	Undesirable Properties	Ref.
*Nigella sativa* (seed)	Thymoquinone (Essential Oil), carbohydrates, proteins, alkaloids, vitamins, minerals	Analgesic, anti-inflammatory, anticancer, antibacterial, antifungal, antioxidant, wound healing	Easy to degrade, volatile	[9]
*Satureja mutica* (seed)	Thymol, carvacrol	Antibacterial, antioxidant	Easy to degrade, volatile	[10]
*Salvia hispanica* L. (seed)	Xylose, glucose, methyl glucuronic acid	Nutritional benefits	/	[11]
Flax (seed)	Polysaccharides, cyanogenic glycosides, cyclic peptides, linolenic acid, cyclic peptides, alkaloids	Antioxidant, free radical scavenger, antibacterial, anti-inflammatory, chronic disease treatment	Poor physical-mechanical properties, profound brittleness, rapid dissolution rate	[12]
Mustard (seed)	Allyl isothiocyanate	Antibacterial, anti-mildew, anti-yeast, food preservation	Strong volatility, heavy smell	[13]
Turmeric (root)	Polyphenols (curcumin)	Anti-inflammatory, anti-oxidant, anti-cancer, analgesic, lowering blood pressure, relieving anxiety, angiogenesis, nerve healing	Poor bioavailability, low water solubility, photodegradation, in vivo instability	[14]
*Rehmannia* (root)	Catalpol (iridoid glycoside)	Neuroprotection	/	[15]
*B. Vulgaris* (root)	Polysaccharide (pectin)	Accelerate wound healing	/	[16]
*Lithospermum erythrorhizon* (root)	Naphthoquinone pigments (shikonin, isobutyl-shikonin, β-hydroxyl-isovaleryl-shikonin, α-methyl-n-butyl-shikonin), quinones	Antibacterial, antioxidant, wound healing, anti-inflammatory	/	[17]
*Blumea balsamifera* (stem)	Flavonoids, L-borneol, caryophyllene, Iedol,D-camphor	Antioxidant, antifungal, antibacterial, anti-tumor	Easy to degrade, volatile	[18]
*Cissus quadrangularis* (stem)	Phytogenic isolated steroid	Anti-osteoporosis, anti-microbial, anti-inflammatory	/	[19]
Pineapple (stem)	Bromelain	Analgesic, anti-inflammatory	/	[20]
*Ziziphus jujuba* (stem)	Terpenoids, tannins, zizipho, tannic acid, flavonoids	Anti-inflammatory, antibacterial, antioxidant, oral treatment	/	[21]
*Zingiber cassumunar Roxb* (rhizome)	Phenylbutanoids, cyclohexene derivatives, naphthoquinones, vanillin, vanillic acid, veratric acid, terpenoids, β-sitosterol, curcuminoids	Anti-bacterial, anti-inflammatory, relieve musculoskeletal pain	Easy to degrade, volatile	[22]
*Dioscorea* (rhizome)	Mucilage, polysaccharide, starch	Neuroprotection, adipocyte aquaglyceroporin modulation, antioxidant, fungicide, amnesia amelioration activities	/	[2]
*Agrimonia eupatoria* L. (aerial parts)	Tannins, flavonoids, phenolic acids, triterpenoids	anti-inflammatory, antioxidant, antimicrobial, antibiofilm properties	/	[23]
*Scutellariae* (radix)	Flavonoid glycoside baicalin, wogonoside	Anti-inflammatory, antioxidant	Poor water solubility	[24]
*Aloe vera* (leave)	Soluble sugars, anthraquinones, polysaccharides, sterols, amino acids, salicylic acid, vitamins, proteins, minerals	Antibacterial, antiviral, antifungal, anti-inflammatory, antioxidant, immune regulation, hypoglycemic, wound healing	Limited by external factors, lack of electrical sensitivity and mechanical properties	[25]
Thyme (leave)	Thymol, carvacrol	Anti-inflammatory, antibacterial, antioxidant, insecticidal, low toxicity	Easy to degrade, volatile	[26]
*Azadirachta indica* (leave)	Azadirachtin	Anti-ulcer, antibacterial, anti-inflammatory, antimalarial, antifungal, antiviral, antioxidant, antimutagenic and anticancer	/	[27]
*Mikania micrantha* (leave)	Deoxymikanolide, scandenolide, dihydroscandenolide, mikanolide, dihydromikanolide, m—methoxy benzoic acid	Antitumor, Antifungal, Cytotoxic, Analgesic, Antibacterial, Antioxidant, Antiviral	/	[28]
*Falcaria vulgaris* (leave)	Carvacrol	Antibacterial, antioxidant	/	[29]
*Moringa oleifera* Lam. (leave)	Quercetin, kampeferol, ascorbic acid (polyphenolic compounds)	Antioxidant properties, retard oxidative stress, and their degenerative effect	Light and oxygen sensitive	[30]
Henna (leave)	Lawsone (2-hydroxy-1,4- naphthoquinone)	Antioxidant, analgesic, anti-inflammatory, antibacterial, antifungal, anticancer activities, healing burns	/	[31]
*Plantago australis* Lam. (leave)	Verbascoside	Antiviral, antimicrobial, anti-inflammatory, antiulcer, antidiarrheal	/	[32]
*Zataria multiflora* (leave)	Phenolic compounds (carvacrol, thymol)	Antispasmodic, Anti-Nociceptive, Antioxidant, Anti-Inflammatory, Antifungal, Antibacterial	/	[33]
*Morinda citrifolia* (leave)	Phenols, alkaloids, terpenes, flavonoids, tannins, caprylic acid, L-asperuloside, caproic acid	Anti-bacterial, anti-helminthic, anti-viral, analgesic, anti-fungal, anti-inflammatory, hypotensive, immune-enhancing	/	[34]
Clove (bud)	Eugenol, eugenol acetate, β-caryophyllene, β-cartilene	Fungi, antioxidant, analgesic, anesthetic, insecticide	Easy to degrade, volatile	[35]
*Calendula officinalis* (flower)	Phenolic compounds, triterpenoids, steroids, terpenoids, carotenes, fatty acids, essential oils, carbohydrates, quinones, minerals, tocopherols, saponins	Anti-inflammatory, antioxidant, antibacterial, antifungal, antiviral properties, blood clotting activity, immunomodulatory	/	[36]
*Althea Officinalis* (petal)	Pectin, starch, flavonoids, sucrose, phytosterol, tannin, amino acids	Antibacterial, anti-inflammatory	/	[37]
*Atropa belladonna* (fruit)	Tropane alkaloids, atropine, hyoscyamine, scopolamine, anisodamine	Antioxidant, anticancer properties	/	[38]
*C. carandas* (fruit)	Phenolics, flavonoids, vitamin C	Anti-inflammatory, antioxidant, antibacterial	/	[39]
*Citrullus colocynthis* (fruit)	Glycosides, flavonoids, alkaloids, curcurbitacins, colocynth oxides, fatty acids, essential oils	Antioxidant, cytotoxic, antidiabetic, antilipidemic, insecticide, antimicrobial, anti-inflammatory	/	[40]
*Ganoderma lucidum* (spore)	*Ganoderma lucidum* triterpenoids	Anti-cancer, anti-inflammatory, anti-oxidation, anti-proliferation	Easy to be oxidized	[41]
*Poria cocos* (mushroom)	Poricoic acid A	Prebiotic functions, diuretic, anti-osteoporosis, anti-inflammatory, anti-tumor	/	[42]
Cinnamon (bark)	Phenol compound	Antibacterial, anti-tumor, antioxidant	Easy to degrade, volatile	[43]
*Pinus halepensis* (bark)	Anthocyanin, phenolic acid	Antioxidant, antiviral, analgesic, cytotoxic, anti-inflammatory, antibacterial	/	[44]
Magnoliae (cortex)	Magnolol	Anti-tumor, anti-inflammatory, antibacterial, antioxidant, antiplatelet, antiarrhythmic	/	[45]
Pomegranate (peel)	Tannins, alkaloids, flavonoids, organic acids	Antioxidant, antifungal, antimicrobial	Easy to oxidize, difficult to absorb	[46]
Oregano (herb)	Rosmarinic acid (phenolic compounds)	Antioxidant, antibacterial, antifungal, sweating, antispasmodic, analgesic	Easy to degrade, volatile	[47]
*Oliveria decumbens* (plant)	Thymol, carvacrol	Antibacterial, antioxidant	Easy to degrade, volatile	[10]
*Frankincense* (tree)	Monoterpenes, diterpenes, lipophilic pentacyclic triterpene acids, polysaccharides, volatile oil	Anti-inflammatory, treatment of inflammatory diseases	Poor oral bioavailability	[48]
Black pepper	Essential oil, limonene	Antibiosis	Easy to degrade, volatile	[49]
Wolfberry	*Lycium barbarum* polysaccharides (polysaccharides), betaine, zeaxanthin, β-carotene, vitamins, amino acids, trace minerals	Anti-aging, anti-tumor, antioxidant and immune regulation, neuroprotection	/	[50]
*Spinacia oleracea*	Multivitamins, minerals, bioflavonoids, chlorophyll, quercetin	Anti-proliferation, anti-oxidation, anti-inflammatory, central nervous system inhibition, liver protection, anti-allergy, anti-γ radiation, anti-osteoporosis	/	[19]
*Cayenne pepper*	Capsaicin (8-Methyl-N-vanillyl-6-nonenamide)	anti-microbial, anti-virulence	/	[4]
Honey	Methylglyoxal	Antibacterial, anti-inflammatory, antioxidant	/	[51]
*Elaeagnus Aangustifolia*	β-carboline alkaloids, cardiac glycosides, esters, flavonoids, phenols, phenolic acids, ketones, phenyl ethers, pyrimidines, steroids, terpenes, vitamins	Analgesic, antibacterial, anti-inflammatory, antioxidant, therapeutic for rheumatoid arthritis and osteoarthritis	/	[52]
*Herba epimedii*	Icariin (flavonoids)	Angiogenesis, anti-osteoporosis, anti-inflammation	/	[53]
*Tripterygium wilfordii Hook F*.	Celastrol (quinone methide triterpene)	Antioxidant, anti-inflammatory	/	[54]
*Salvia miltiorrhiza*	Tanshinone, Danshen acid	Antioxidant, anti-inflammatory, anti-hypoxia, anti-arteriosclerosis, anti-apoptosis	Poor water solubility, low bioavailability, instability	[55]
Amla oil	Vitamin(A/C), fiber, mineral (potassium, magnesium, calcium)	Antioxidation, enhance bone mineralization	Easy to degrade, volatile	[56]
*Centella asiatica*	Asiatic acid, asiaticoside, madecassic acid, madecassoside(triterpenoid)	Heal wounds, burns, ulcerous abnormalities of the skin, stomach and duodenal ulcers, leprosy, lupus, scleroderma, diseases of the veins	/	[57]
*Astragalus membranaceus*	Polysaccharides, saponins	Anti-inflammatory, immunoregulatory, stimulating cell metabolism, lowering blood sugar, promoting cell proliferation	Poor water solubility	[58]
*Angelica gigas Nakai*	Decursin, decursinol angelate, decursinol	Anti-cancer	Poor water solubility	[59]
Ferula gum	resin, essence, gum, free ferulic acid, vanillin	Antibiosis	/	[20]
*Cirsium Japonicum DC*	Total flavonoid, essential oil, lipid, saccharides	Hemostasis, anti-inflammatory, antitumor	/	[60]
*Quilaja saponaria*	Saponin	Growth Promotion, Antimicrobial, Insecticidal	/	[61]
*Yerba mate*	Polyphenol (xanthines, saponin, caffeoyl derivatives)	Antioxidant, cholesterol-lowering, liver, diuretic, regulate blood sugar, stimulate the central nervous system	Easy thermal degradation	[62]
*Helicid nilgrinica Bedd*	Helicid	Treatment of insomnia and headache	Poor water solubility	[63]

**Table 2:** Comparison of advantages and disadvantages between electrospinning and other technologies.

Technology	Driving Force	Merit	Defect	Ref.
Electrospinning Technique	Electrostatic force	Top-down integrated process with small and uniform fiber diameter, high porosity and large surface area	Narrow range of materials, high voltage safety issues, relatively low productivity	[140]
Stretch	Stretching force	Simple process, low cost, thin fiber diameter, high crystallinity, good alignment and enhanced tensile strength	Complex equipment, fixed structure, cannot change with the process and material changes	[162]
Self-assembly	Evaporation/Van der Waals force	Various functional and active building blocks can be integrated to obtain complex 3D structures with high fault tolerance	For biomolecules, high cost, polydispersity and purification limitations, lack of stability in nonaqueous solutions and at high temperatures	[163]
Phase separation	Centrifugal force	Good crystallinity and tensile properties	Centrifugal dispersion operation easily affects morphology	[164]
Template synthesis	/	Wide range of materials available, nanomaterials with clearly defined size, shape and structure	Fiber damage during template removal	[165]
Melt spraying	High temperature extrusion	Short process flow, high production efficiency, fine and uniform fibers	High energy consumption, poor fiber orientation, low web strength	[166]
Freeze drying	Lyophilization	Maintains original chemical composition and physical properties to improve substrate instability	Cost high	[167]
Solvent casting	Evaporation drying	Able to prepare ultra-thin films with high optical transparency and rapidly degradable porous films	Organic solvents cause health and environmental problems	[168]
Laser ablation	Laser high energy	Green simple process, wide selection	Low yield	[169]
Chemical vapor deposition	/	High yield, fiber diameter, crystallinity and orientation can be tailored by precisely controlling the synthesis conditions	Discontinuous short fibers	[170]
Blown spinning	Air compression	Can be applied to any surface or substrate to overcome the limitations of electronic spinning on in-situ synthesis and conductive targets	Small surface area and poor morphology of fiber	[171]
Carbon Dioxide (CO_2_) Laser Supersonic Drawing	Supersonic stretching	Uniform diameter, continuous nanofibers, solvent-free, high efficiency	For polymers with good thermoplasticity only	[172]
Force spinning technique	Centrifugal force	Increased productivity, increased material selection and reduced fiber costs	Complex structure and heavy load of production equipment	[173]
Dry-wet spinning	/	Spinning solution with high viscosity, low solvent recovery and consumption, fast forming speed, uniform fiber structure, high strength and elasticity	After the spinning solution is broken, it is easy to flow along the spinneret	[174]
Electrohydrodynamic (EHD) printing	Electrostatic force	Pattern fiber materials by digitally controlled material deposition (usually layer by layer) to create ordered freeform geometries	/	[175]

**Table 3:** Characteristics of polymer nanofibers synthesized by electrospinning herbal medicine.

Synthetic Polymer	Doped Polymer	Solvent	Herbal Extract	Electrospinning Technique	Characteristic	Ref.
PCL	/	DCM/DMF	Icariin	Blend	Cell penetration, collagen deposition and angiogenesis with bone regeneration potential	[191]
	/	Chloroform/Methanol	*Aloe vera*	Blend	Can support tissue and has a controlled surface morphology and structure to treat different skin conditions or injuries	[192]
	/	/	Eugenol	Blend	Promote the production of collagen	[35]
	/	DCM/DMF	*Acalypha indica* leaf extract	Blend/Post-treatment	Microbial inhibition to keep food fresh	[193]
	/	DCM/DMF	Shikonin	Blend	Loading and controlling the release of bioactive components has great potential in the treatment of wound healing or atopic dermatitis	[194]
	/	THF/Methanol	*Cissus quadrangularis* extract	Blend/Post-treatment	Enhanced roughness, mechanical properties and wettability of the scaffold to provide osteoinductive properties in vivo	[195]
PLA	/	DCM	*Azadirachta indicaing* extract/Eucalyptus Citriodora extract	Blend	Biodegradable masks to inhibit bacterial spread for antimicrobial use	[196]
PLLA	/	Chloroform/Methanol	*Cissus quadrangularis* extract	Blend	Enhancing protein absorption and cell viability to induce osteogenic differentiation of MSCs	[184]
PLGA	/	HFIP	*Aloe vera*	Blend	Improved wound closure and re-healing, topical application of high-concentration aloe PLGA-AV nanofibers provides strategy for the treatment of chronic wounds	[197]
	/	HFIP	*Aloe vera*	Blend	Promising Strategies for Treating Chronic Wounds	[107]
PU	/	DMF	Ayurveda oil	Blend	Improved surface roughness and anticoagulant properties with excellent physicochemical and blood compatibility	[56]
	/	DMF	*Nigella sativa* oil	Blend	No cytotoxicity, increased cell viability, synergistically accelerates wound healing	[198]
PVA	/	Deionized water	Honey/Curcumin	Blend	Shows enhanced water management properties and antimicrobial properties for potential wound dressings and tissue engineering	[199]
	/	Distilled water	*Mikania micrantha* extract	Blend	Shows enhanced water management, excellent antibacterial efficiency and biocompatibility	[28]
	/	Ethanol/Pure water	Astragalus polysaccharide/Astragaloside IV	Blend	Synergistic effect, better inhibit inflammation, promote collagen deposition and wound re-epithelialization, conducive to diabetic wounds.	[58]
	/	distilled water/EtOH	Angelica gigas Nakai	Blend	Improved water solubility and rapid dissolution, higher antiproliferative activity, provide a promising method for the treatment of oral cancer	[59]
	/	Water	*Azadirachta indica* extract	Blend	Enhanced water management and thermal performance for bacterial inhibition	[200]
	/	Ethanol/distilled water	*Plantago australis* Hydroethanolic extract	Blend	Promote cell proliferation and accelerate wound closure	[32]
	/	DMSO	*Thespesia populnea* extract	Blend	Better drug release, extended activity, excellent antibacterial activity without hindering cell growth	[81]
	/	/	*Cissus quadrangularis* extract/*Galinsoga parviflora* Cav extract	Blend	Sufficient tensile properties and excellent antibacterial activity, cell viability, blood compatibility	[201]
	/	double-distilled water	*Morinda citrifolia* extract	Blend	Nanostructured morphology that interacts effectively with protein GSK3β binding sites to enhance cell proliferation and adhesion	[34]
	/	/	Coptidis Rhizoma extract	Blend	High drug loading efficiency and sustained release with high antibacterial activity	[202]
PVP	/	Ethanol	Isatis root extract	Blend	Good air permeability, wettability, antibacterial properties and faster wound healing	[120]
	/	Ethanol/Deionized water/DMF	*Aloe vera*/*Aloe vera* acetate	Blend	Good compatibility, thermal stability, hydrophilicity, tensile strength, this hydrated material is conducive to application in tissue engineering scaffolds	[203]
	/	Ethanol/DMAc	Helicide	Tri-fluid side-by-side	Fast, efficient and easy delivery of actives	[204]
PEO	PCL	Ethanol/Glacial acetic acid	*Ganoderma lucidum* triterpenoids/Methotrexate	Coaxial	Chinese and Western medicine successfully encapsulated and uniformly dispersed in the polymer matrix, with biphasic release characteristics, synergistic inhibition of HeLa cells	[41]
EE100	/	Ethanol	Oregano ethanolic extract	Blend	High stability and sustained release	[47]
Nylon66	/	Formic acid	*B.vulgaris* extract	Blend	Promote the adhesion, proliferation and differentiation of H-keratin and MSCs into epithelial lineage	[16]
Carbomer	PAN	DMF	*Ziziphus jujuba* extract	Blend	Controlled release, improved anti-inflammatory function, for periodontal disease, improve patient compliance	[21]
PHA	/	DCM	Plai oil	Blend	Enhanced mechanical strength, non-toxic and cell-attached, available with the development and design of pain-relieving transdermal patch products	[22]

**Table 4:** Characteristics of electrospun herbal medicine natural polymer nanofibers.

Natural Polymer	Doped Polymer	Solvent	Herbal Extract	Electrospinning Technique	Characteristic	Ref.
Chitosan	/	Glacial acetic acid/Double-distilled water	Honey/Capsaicin extract	Blend	High levels of cell viability and proliferation and better wound closure	[4]
Hyaluronic acid	/	Distilled water/DMSO	curcumin/usnic acid	Blend	Preparation of Biocompatible Nanofibers Without Other Carrier Polymers and Catalysts	[213]
Cellulose acetate	/	Acetone/DMAc	Asiaticoside	Blend	Greater water retention and low toxicity, with potential for topical/transdermal or wound dressing patches	[57]
	/	Acetone/DMF	*Blumea balsamifera* oil	Blend	Biphasic release, good wound permeability, sufficient biocompatibility and high efficacy inhibit bacterial growth	[18]
	/	Acetone/DMAc	Asiaticoside/Curcumin	Blend	Antioxidant activity to support fibroblast adhesion and proliferation	[108]
	/	Acetone	*Acanthus ebracteatus* Vahl. extract	Blend	Antioxidant activity, reduced extract cytotoxicity, potentially for local delivery of bioactive components	[82]
	/	Acetone/DMF/Water	Quillaja saponin	Blend	Good antifungal activity	[61]
Arabinose	Gelatin/PVA/PVP	Deionized water/Ethanol	*Aloe vera*/*Trachyspermum ammi* essential oil	Coaxial	Enhancing wound closure and eliminating bacterial infection at the wound site	[221]
Guar gum	PVA	Distilled water	*Acalypha indica*/*Aristolochia bracteolata*/*Lawsonia inermis*/*Thespesia populnea*	Blend	Enhances wound contraction, fully forms the dermis and epidermis, and has smaller scars by expressing stem cells and wound healing-related genes	[220]
Pullulan	Chitosan	Deionized water	*Opuntia cochenillifera* extract/Cactaceae extract	Blend	Demonstrated water absorption, enhanced thermal stability and good bacterial inhibition and biocompatibility	[236]
Sodium alginate	CA/PEO	Acetone/Water	*Pinus halepensis* bark extract	Blend	Eliminates skin inflammation, reduces patient discomfort and can be used for radiation dermatitis treatment	[224]
Dextran	Zein	Acetic acid/Water	Curcumin	Blend	Effective free radical scavenging activity and iron reducing ability, and controlled release behavior required for curcumin delivery	[227]
Xanthan gum	Chitosan	Formic acid	Curcumin	Blend	High entrapment efficiency, physical stability in aqueous media and long-term pH-stimulated release	[225]
Collagen	Poly(3-hydroxybutyric acid/Gelatin	HFP	*Coccinia grandis* extract	Blend/Post-treatment	Improved tensile strength and increased wound epithelial regeneration	[228]
Gelatin	/	Ethanol/Acetic acid	Crude *C. carandas* fruits ethanol extract	Blend	It has higher thermal stability and high antioxidant activity, and has application potential in cosmetic masks.	[39]
	/	Acetic acid/Double-distilled water	Ethanolic pomegranate peel extract	Blend	High encapsulation efficiency and increased bioavailability of bioactive compounds for pharmaceutical or food applications	[237]
	/	/	*Centella asiatica* extract	Blend	Biodegradable, promotes fibroblast proliferation and collagen synthesis, and exhibits antibacterial activity	[238]
Silk fibroin	PEO	Glacial acetic acid	Cirsium Japonicum DC	Blend	Chinese herbal medicine ingredients significantly improve cell compatibility and hemostatic performance of nanofiber matrix	[60]
Zein	/	Glacial acetic acid/Ethanol	*Laurus nobilis* essential oil/*Rosmarinus officinalis* essential oil	Blend	Prominent antibacterial activity, more sustained release, suitable for edible active food packaging	[235]
Keratin	PCL/PEO/CS	Formic acid/Acetic acid	*Aloe vera* extracts	Coaxial	Increased tensile strength, showing biocompatibility and improved adhesion	[206]

**Table 5:** Antibacterial types of herbal medicines.

Drug Plant	Families Genera	Extraction Method	Use Ingredients	Inhibiting Flora	Ref.
*Aloe vera*	Liliaceae	/	Aloe gel	*Staphylococcus aureus*/*Staphylococcus epidermidis*	[206]
*Curcuma longa* L.	Turmericaceae	Acid hydrolysis	Curcumin	*Bacillus cereus*/*Escherichia coli*/*Salmonella typhimurium*/*Staphylococcus aureus*	[14]
*Azadirachta indica*	Meliaceae	Methanol, Acetyl and Water	Azadirachtin	*Staphylococcus aureus*	[200]
*Calendula officinalis*	Composite family	Ethyl alcohol	/	*Staphylococcus aureus*/*Escherichia coli*	[36]
*Centella asiatica*	Umbelliferae	/	Asiaticoside	/	[57]
*Lawsonia inermis* (Henna)	Lythraceae	Water/ethanol	Lawsone	*Staphylococcus aureus*/*Escherichia coli*	[223]
*Zataria multiflora*	Lamiaceae	/	/	*Candida albicans*/*Pseudomonas aeruginosa*/*Staphylococcus aureus*	[33]
*Halepensis*	Coniferous species	Water	/	/	[44]
*Honeyor propolis*	/	/	/	*Staphylococcus aureus*/*Escherichia coli*/*Pseudomonas aeruginosa*/*Candida albicans*	[51]
*Lithospermum erythrorhizon* (Lithospermi radix)	Borage family	Methanol	Shikonin	*Staphylococcus aureus*/*Escherichia coli*	[231]
*Nigella sativa*	Ranunculaceae	Cold pressing	/	*Staphylococcus aureus*/*Escherichia coli*	[9]
Clove	Olive family	/	/	*Escherichia coli*/*Staphylococcus aureus*, and *Pseudomonas aeruginosa*	[208]

## Data Availability

Not applicable.
